# SAM‐dPCR: Accurate and Generalist Nuclei Acid Quantification Leveraging the Zero‐Shot Segment Anything Model

**DOI:** 10.1002/advs.202406797

**Published:** 2024-12-27

**Authors:** Yuanyuan Wei, Shanhang Luo, Changran Xu, Yingqi Fu, Yi Zhang, Fuyang Qu, Guoxun Zhang, Yi‐Ping Ho, Ho‐Pui Ho, Wu Yuan

**Affiliations:** ^1^ Department of Biomedical Engineering The Chinese University of Hong Kong Shatin Hong Kong SAR China; ^2^ Department of Biomedical Engineering National University of Singapore Singapore Singapore; ^3^ Department of Computer Science and Engineering The Chinese University of Hong Kong Shatin Hong Kong SAR China; ^4^ Department of Electronic Engineering The Chinese University of Hong Kong Shatin Hong Kong SAR China; ^5^ Department of Automation Tsinghua University Beijing 100084 China; ^6^ Centre for Biomaterials The Chinese University of Hong Kong Hong Kong SAR China; ^7^ Hong Kong Branch of CAS Center for Excellence in Animal Evolution and Genetics Hong Kong SAR China; ^8^ State Key Laboratory of Marine Pollution City University of Hong Kong Hong Kong SAR China

**Keywords:** deep‐learning, digital PCR, droplet microfluidics, nucleic acid quantification, segment anything model

## Abstract

Digital PCR (dPCR) has transformed nucleic acid diagnostics by enabling the absolute quantification of rare mutations and target sequences. However, traditional dPCR detection methods, such as those involving flow cytometry and fluorescence imaging, may face challenges due to high costs, complexity, limited accuracy, and slow processing speeds. In this study, SAM‐dPCR is introduced, a training‐free open‐source bioanalysis paradigm that offers swift and precise absolute quantification of biological samples. SAM‐dPCR leverages the robustness of the zero‐shot Segment Anything Model (SAM) to achieve rapid processing times (<4 seconds) with an accuracy exceeding 97.10%. This method has been extensively validated across diverse samples and reactor morphologies, demonstrating its broad applicability. Utilizing standard laboratory fluorescence microscopes, SAM‐dPCR can measure nucleic acid template concentrations ranging from 0.154 copies µL^−1^ to 1.295 × 10^3^ copies µL^−1^ for droplet dPCR and 0.160 × 10^3^ to 3.629 × 10^3^ copies µL^−1^ for microwell dPCR. Experimental validation shows a strong linear relationship (r^2^ > 0.96) between expected and determined sample concentrations. SAM‐dPCR offers high accuracy, accessibility, and the ability to address bioanalytical needs in resource‐limited settings, as it does not rely on hand‐crafted “ground truth” data.

## Graphical abstract

SAM‐dPCR, a training‐free, open‐source method is introduced for rapid and accurate nucleic acid quantification. Leveraging the zero‐shot Segment Anything Model (SAM) and a fluorescence‐intensity‐responsive classifier, SAM‐dPCR achieves over 97.10% accuracy in analyzing over 2,000 reactors for each sample. SAM‐dPCR offers high accuracy, accessibility, and the ability to address bioanalytical needs in resource‐limited settings, eliminating hand‐crafted ‘ground truth’ data.

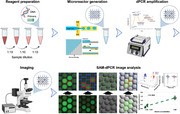

## 1 Introduction

Since life emerged approximately 3.5 billion years ago, nucleic acids have been the cornerstone of genetic information, acting as the blueprint for all living organisms. Their complex structures and vital functions have spurred scientific research, leading to significant discoveries that unravel the origins and intricacies of life. In contemporary times, the rising incidence of infectious diseases globally, coupled with the relentless threat of the COVID‐19 pandemic, which has affected over 711 million people worldwide (704753890 confirmed cases and 7010681 deaths) as of April 2024,^[^ ^1^ ^]^ emphasizes the critical need for rapid and precise nucleic acid quantification.^[^ ^2^, ^3^, ^4^ ^]^ Rapid and accurate molecular diagnostics are essential in combating global pandemics of infectious diseases^[^ ^5^ ^]^ such as malaria,^[^ ^6^ ^]^ dengue,^[^ ^7^ ^]^ tuberculosis,^[^ ^8^ ^]^ and monkeypox.^[^ ^9^ ^]^ Given that each mammalian cell contains merely about 6 picograms of DNA, the necessity for ultrasensitive detection methods has never been more evident.

An enabling technology is digital Polymerase Chain Reaction (dPCR), facilitating the detection and quantification of rare mutations and target sequences.^[^ ^10^ ^]^ Unlike conventional polymerase chain reactions (PCR) or quantitative PCR (qPCR) – which are based on relative quantification – dPCR affords high‐sensitivity absolute quantification of nucleic acids.^[^ ^11^, ^12^ ^]^ Confining PCR reactions in monodispersed picoliter microreactors mitigates the amplification bias and boosts reproducibility.^[^ ^10^, ^13^ ^]^ However, it also introduces challenges, including high cost, complexity, lengthy processing time, and potential error vulnerability.^[^ ^14^, ^15^, ^16^ ^]^ In dPCR, samples are distributed into thousands of compartments for amplification.^[^ ^12^, ^17^, ^18^ ^]^ The inferred concentrations are determined via Poisson statistics to analyze the ratio of positive (fluorescent) to negative (non‐fluorescent) droplets.^[^ ^19^, ^20^ ^]^ Accurate identification of positive droplets in dPCR images thus ensures the reliability of nucleic acid quantification. However, traditional detection methods such as commercialized machines, flow cytometry, and manual fluorescence imaging, reveal significant limitations. Commercialized dPCR platforms such as those from Bio‐Rad and Raindance, while precise, pose challenges including the cost of specialized facilities and the need for operational expertise. High initial and recurring expenses may deter use, especially in budget‐strapped labs.^[^ ^19^, ^20^ ^]^ While flow cytometry stands as a revered “gold standard” for cell analysis and sorting, it is less practical for the high‐throughput needs of dPCR due to its high costs, the necessity for expert operation, and complex sample preparation.^[^ ^21^ ^]^ Similarly, fluorescence imaging, dependent on software or manual counting, is laborious and susceptible to errors, further highlighting the limitations of these methods for routine dPCR use where large sample volumes and swift analysis are crucial.^[^ ^22^ ^]^ Automated counting and dynamic thresholding struggle with calibration, particularly with diverse samples or weak signals, leading to quantification errors that affect the precision of nucleic acid detection in key dPCR processes.^[^ ^21^ ^]^

Recent advancements in bio‐analysis have significantly benefited from the integration of artificial intelligence (AI), enhancing data analysis capabilities.^[^ ^23^, ^24^, ^25^, ^26^, ^27^ ^]^ Neural networks, with their end‐to‐end learning and adaptive capacities, align perfectly with the demands of dPCR image analysis. ^[^ ^28^ ^]^ By eliminating explicit feature engineering and adopting automated learning, they streamline the process, effectively managing complex datasets and optimizing analytical procedures, addressing cost, methodological complexities, processing time, and error susceptibility. For instance, as the first case where deep learning was applied to droplet dPCR (ddPCR) image analysis, the model combined with circle Hough transform (CHT) has demonstrated robust performance in quantifying pathogenic bacteria (*Escherichia coli* O157:H7), from out‐of‐focus images with a detection limit of 4.0 × 10^1^ copies µL^−1^.^[^ ^29^ ^]^ Another example presents a deep learning‐based framework combining Hough transform and convolutional neural networks (CNNs) for high‐throughput ddPCR image analysis, achieving 99.71% accuracy in positive droplet recognition. The method effectively filters out invalid droplets and performs robust detection under varying conditions without requiring image pre‐processing or normalization.^[^ ^30^ ^]^ Another model, YOLOv3, attained a 98.98% accuracy while reducing labeling time by 70% compared to Mask R‐CNN and YOLO models.^[^ ^31^ ^]^ In addition to ddPCR, the neural networks' robust generalization capability has also been validated across various dPCR formats. The Deep‐qGFP model demonstrated 96.23% accuracy and a detection speed of 2.5 seconds per image on droplet‐based, microwell‐based, and agarose‐based dPCR image datasets.^[^ ^32^ ^]^ However, supervised models require extensive hand‐crafted data or “ground truth” for training, leading to exhaustive data collection and manual annotation.^[^ ^33^, ^34^ ^]^ This often results in a significant gap between training and testing domains due to the inherent variability. Additionally, the need for interframe continuity in these supervised methods may limit their ability to adapt to rapid transformations, especially when there are subtle differences between droplet populations or under non‐ideal imaging conditions.^[^ ^35^, ^36^ ^]^ This limitation necessitates further model training to adapt to new experimental settings, thereby affecting the comparability and reproducibility of results across different laboratories and platforms.

To address these challenges, we introduce SAM‐dPCR, a training‐free open‐source pipeline for accurate and high‐throughput bioanalysis. Pre‐trained on over 1 billion masks from 11 million images, the “Segment Anything Model” (SAM) by Meta offers adaptability without the need for extensive retraining or fine‐tuning.^[^ ^37^ ^]^ SAM‐dPCR integrates the zero‐shot SAM and a training‐free fluorescence‐intensity‐responsive classifier with dPCR's efficiency and precision. SAM‐dPCR converts dPCR images into 3D maps, revealing target molecule distributions and concentrations. We tested SAM‐dPCR on various nucleic acid samples, including ddPCR generated by a customized microfluidic chip and microwell dPCR generated by a commercialized machine. To assess the reliability and accuracy of our model, we determined its Limit of Quantification (LoQ) and Limit of Detection (LoD) by testing on datasets acquired under varied experimental conditions. The LoQ for ddPCR ranged from 0.154 copies µL^−1^ to 1.295 × 10^3^ copies µL^−1^. For microwell dPCR, it was between 0.160 × 10^3^ to 3.629 × 10^3^ copies µL^−1^. LoD evaluation involved analyzing mask counts per image, acceptable input image sizes, and performance on different imaging conditions (imaging modes and signal‐to‐noise ratios, namely SNRs). Additionally, we compared SAM‐dPCR with other state‐of‐the‐art (SOTA) models such as CNNs^[^ ^30^ ^]^ and Deep‐qGFP.^[^ ^32^ ^]^ Our findings lay the groundwork for investigating the potential use of SAM‐dPCR in molecular biology research.

## 2 Results

**Figure 1:**
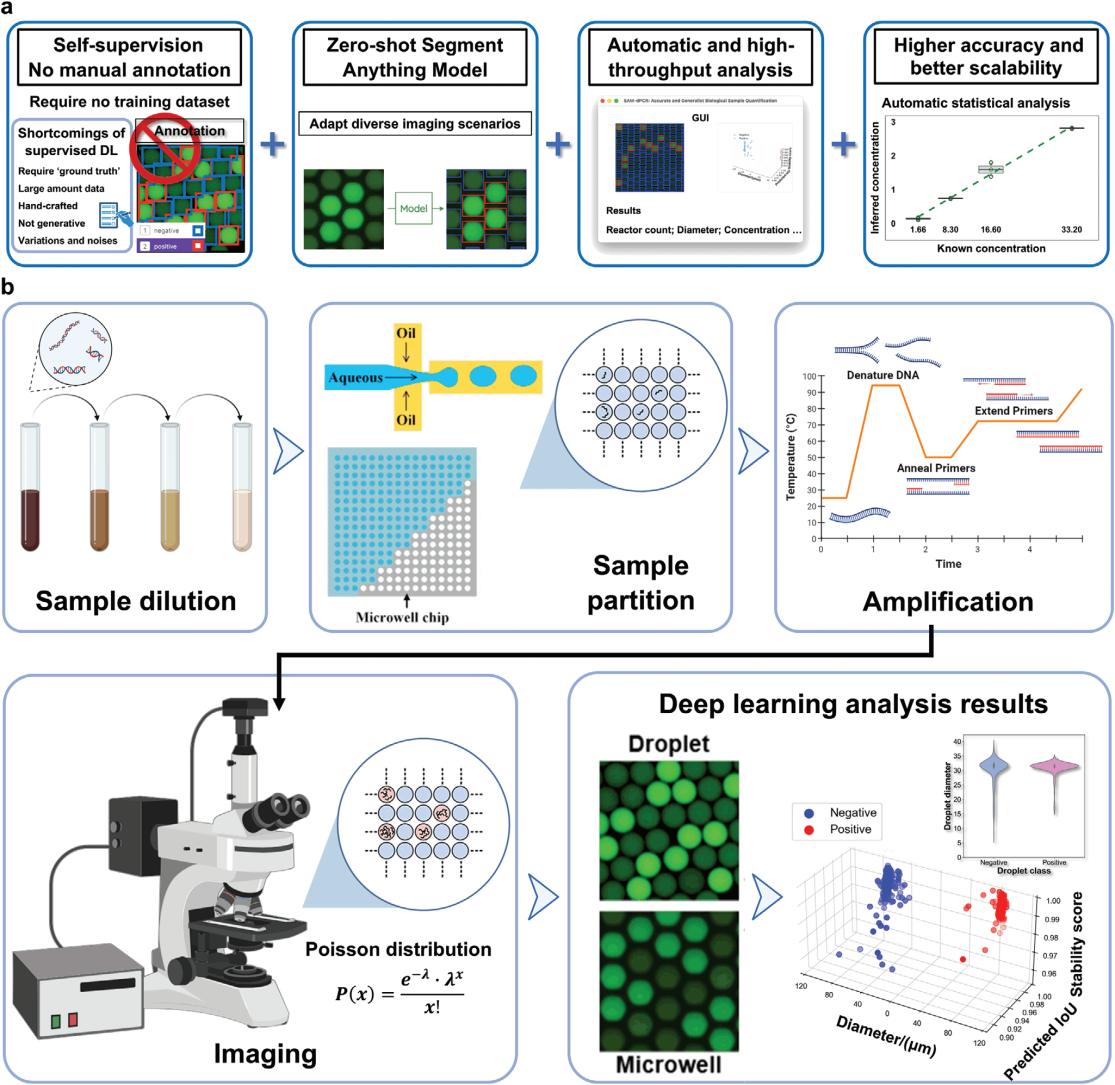
Operational procedure of open‐source SAM‐dPCR platform for nucleic acid analysis. Process of sample dilution, partition, amplification, imaging, analysis (microreactor segmentation and classification), and final template concentration estimation applied in dPCR analysis domains. This is accomplished by incorporating the zero‐shot SAM and dPCR. SAM's potential is further amplified and validated, providing real‐time and informative data visualization of each microreactor in both droplet and microwell domains.

Designed for accurate and high‐throughput absolute quantification, the SAM‐dPCR paradigm effectively quantifies target nucleic acid templates without the necessity for annotated training data. As displayed in **Figure** **1**, our samples underwent sequential dilution and partitioning on droplet‐based and microwell‐centered platforms. Utilizing a customized lab‐on‐a‐chip system, each sample produced over 20000 monodispersed droplets, each measuring 46.37 ± 1.64 µm in diameter (equivalent to a volume of 52.20 pL). Post a three‐temperature ddPCR amplification process, droplets were imaged using high‐resolution FITC (Fluorescein isothiocyanate) fluorescence microscopy. We merged droplet counts from 10 images into a single “meta‐droplet” comprising over 2000 droplets, both positive and negative, with each image contributing about 200 droplets. We used random sampling without overlap to ensure equal contribution from each image. Consistent imaging conditions, including uniform focusing, fluorescence intensity, and magnification, were maintained. These captured images were then subjected to real‐time processing through a graphical user interface (GUI, Figure S1, Supporting Information) and put through the SAM‐dPCR algorithm for analysis. To ensure accuracy, trained laboratory personnel annotated the images manually, utilizing *Roboflow* for precise object segmentation and classification. Droplet diameter measurements were conducted using *ImageJ*. To ensure the accuracy and consistency of the ground truth, the annotations were cross‐checked by a separate group of experts who independently reviewed and verified the labels.

In the analysis procedure, droplets were classified as either positive (labeled in red) or negative (labeled in blue) depending on whether they contained templates. The 3D plots include three axes: “diameter/(µm)”, “Predicted IoU” (Intersection over Union), and “Stability Score”. The “diameter/(µm)” is calculated by measuring the droplet diameter in the image data. This value helps characterize the uniformity of droplets since the concentration calculation is related to droplet volume. The “Predicted IoU” is generated automatically by the model to assess the overlap between the predicted segmentation masks and the true regions. A higher IoU value indicates a better match between the predicted and true masks. The “Stability Score” quantifies the consistency of the model's predictions across multiple images, with higher scores indicating more stable and reliable performance. These metrics collectively demonstrate the capability of the SAM‐dPCR model allowing for swift analysis: 3.16 seconds per 1024×1024 pixel dPCR image containing 200∼400 reactors. This level of detail is not achievable with conventional methods or reported AI‐assisted pipelines, which only generate calculated concentration. Our approach also outperforms time‐consuming methods like manual counting or *ImageJ*, which takes up to 30 seconds to process the same number of droplets.

**Figure 2:**
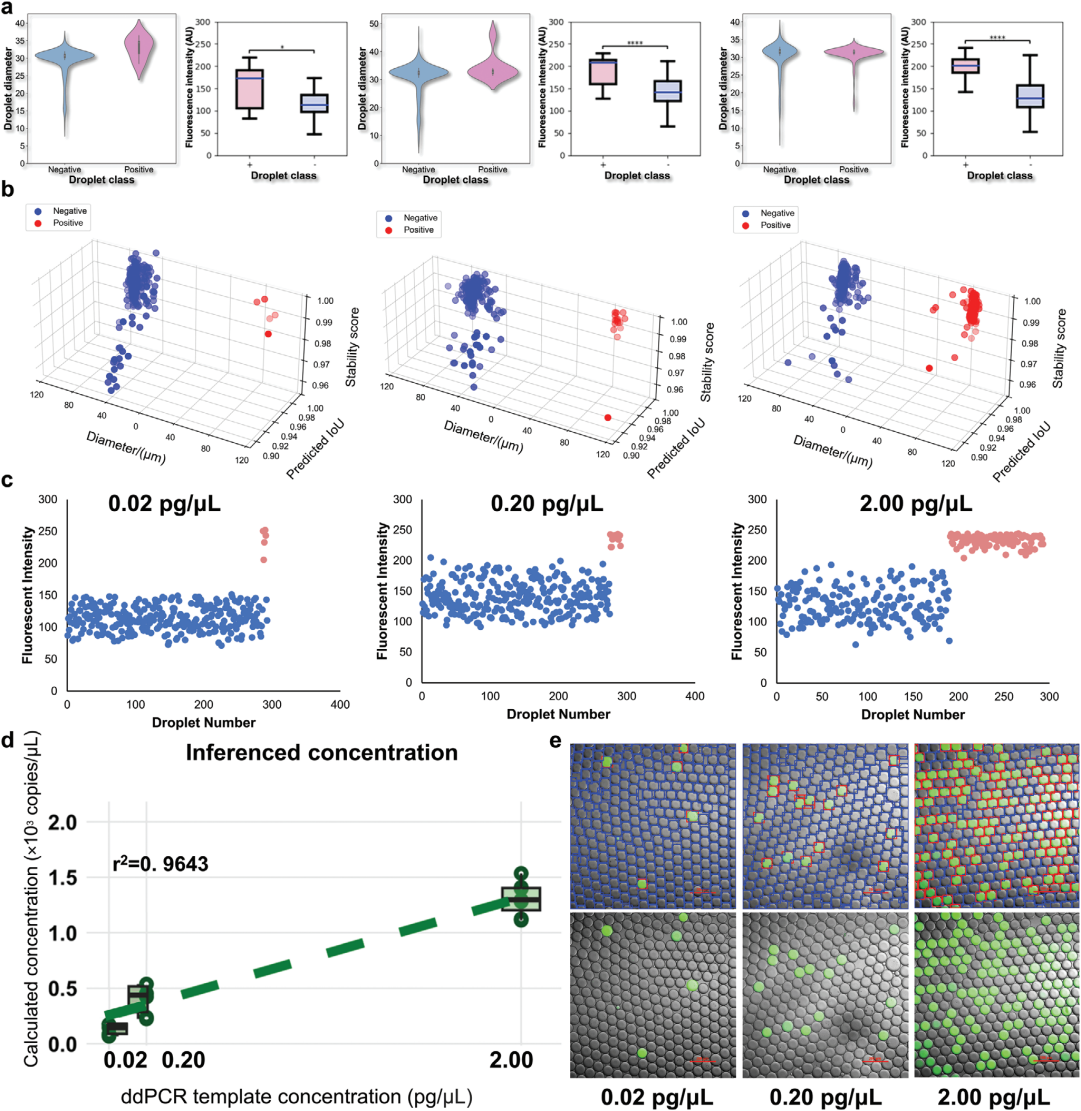
Performance of SAM‐dPCR on droplet dPCR tasks with varying sample concentrations. The figure displays sample images overlaid with masks. The sample images are from benchtop droplet dPCR experiments, with serially diluted templates. The segmentation masks, automatically annotated by SAM, outline the microreactors for further classification and analysis. The statistical analysis of droplet classification and fluorescence intensity further confirmed the validity and accuracy of our method by revealing a significant difference. Sample concentrations are determined by fitting the analysis results to a Poisson distribution. The precision and accuracy of SAM‐dPCR are confirmed through linear regression equations (r^2^ values of 0.96). Scale bar: 200 µm.

**Figure 3:**
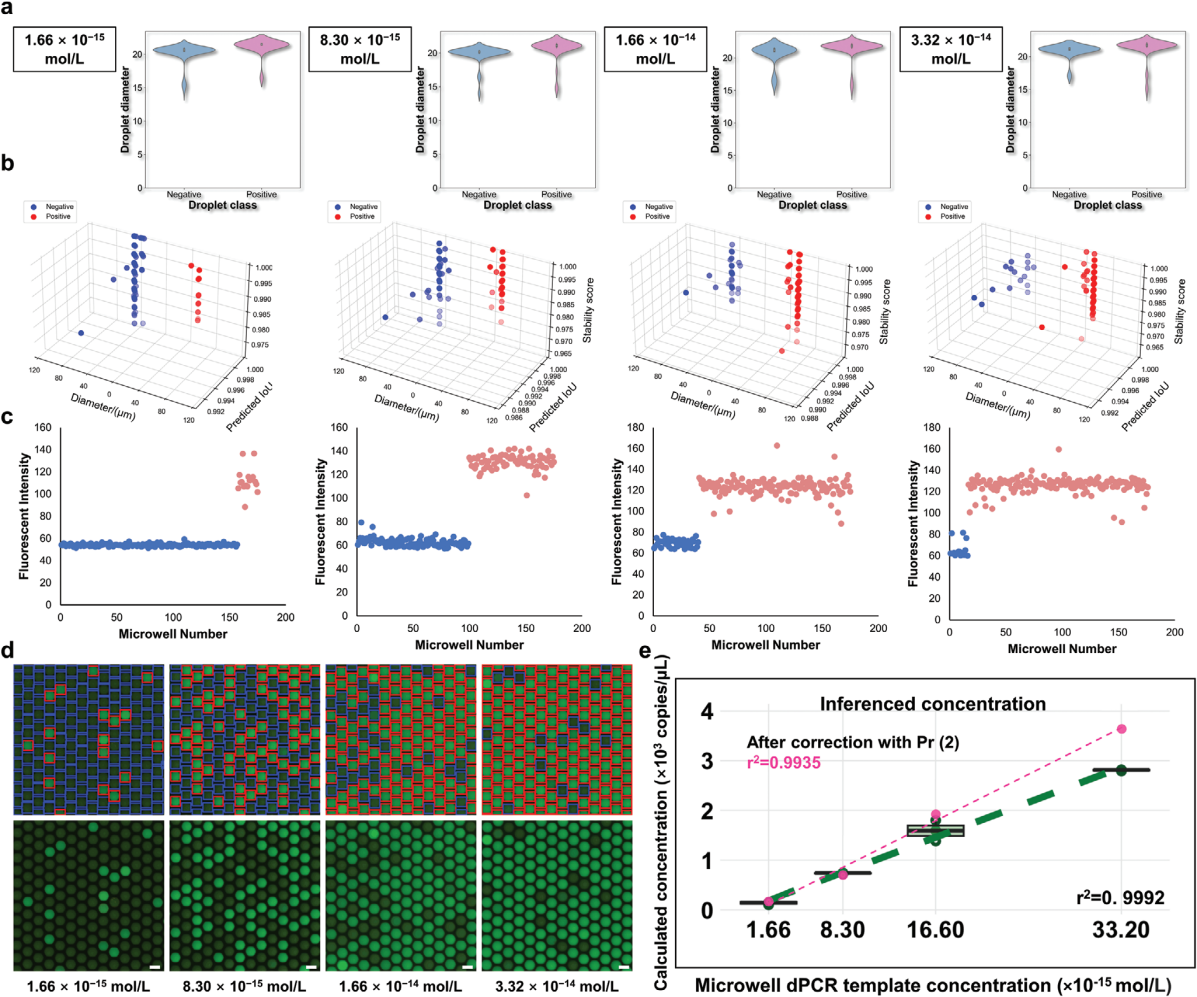
Performance of SAM‐dPCR on microwell dPCR tasks with varying sample concentrations. This figure illustrates SAM‐dPCR's automated analysis of microreactor images from microwell dPCR experiments using serial dilutions. The segmentation masks generated by SAM delineate microreactors for classification and analysis. Concentration estimation utilizes Poisson distribution fitting, with an observed r^2^ value of 0.99, highlighting the method's precision and reliability. Scale bar: 200 µm.

Validations were carried out across various sample concentrations to ascertain the performance of SAM‐dPCR. As illustrated in **Figures** **2**,**3**, applications of SAM‐dPCR across droplet and microwell dPCR testing experiments produced strong linearity between known and inferred concentrations with r^2^ values reaching 0.96 and 0.99, affirming the accuracy and effectiveness of our approach. Here r^2^ represents the coefficient of determination, computed using the standard formula for linear regression. The analysis results yielded inferred concentrations ranging from 0.132 × 10^3^ to 1.295 × 10^3^ copies µL^−1^ and from 0.160 × 10^3^ to 3.629 × 10^3^ copies µL^−1^ for droplet and microwell dPCR testing, respectively. To further assess the reliability of our analysis results, statistical analysis was performed on the correlation between classification results and corresponding fluorescence intensity as depicted in Figure 2. Two out of the three ddPCR images analyzed showed highly significant differences in fluorescence intensity between sample classes, with p‐values below 0.0001. Our validation strategy adopted Seahorse (*Hippocampus kuda*) genome extracts specifically the cytochrome c oxidase subunit I (*COI*) region (206 bp amplicon size), serially diluted to concentrations of 0.4 pg, 4 pg, and 40 pg within a 20 µL PCR system. Similarly, we prepared the microwell dPCR dataset with concentrations spanning 1.66 × 10^−15^ mol L^−1^ to 1.66 × 10^−13^ mol L^−1^, retaining a reaction‐well volume of 755 pL (3D Digital PCR chip v2, ThermoFisher Scientific, USA). Our amplified templates encompassed two double‐stranded blaNDM and blaVIM genes, crucial in regulating the expression of β‐lactamases (bla) – effective carbapenem antibiotics. Ensuring robustness in our results, we examined more than 2000 individual microwells, both positive and negative, distributed across 10 images, each containing around 200 microwells. To further refine the accuracy, we calculated the probabilities of zero ($P_{r}\left(X=0\right)$), single ($P_{r}\left(X=1\right)$), double ($P_{r}\left(X=2\right)$), and multiple ($P_{r}\left(X\geq 1\right)$) copies of the target gene within each microreactor. For high template concentrations (λ > 1), mathematical corrections were applied using $P_{r}\left(X=2\right)$ for the two highest concentrations in microwell dPCR. Detailed calculations and results of these probabilities for each experimental setting are provided in Tables S1 and S2 (Supporting Information). For a more detailed description of the dPCR protocols, please refer to the Materials and Methods section.

**Figure 4:**
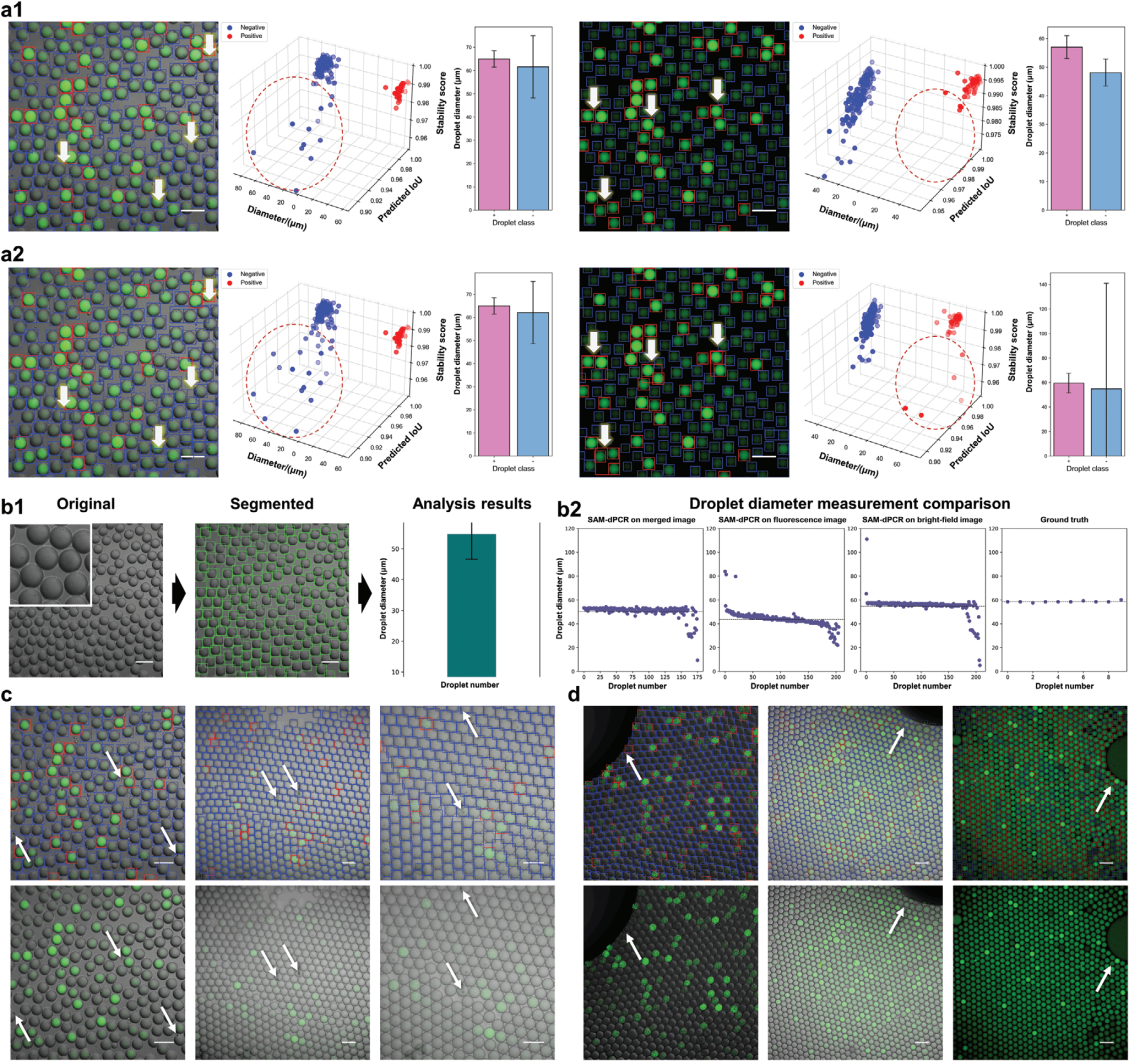
Robustness and versatility of SAM‐dPCR in diverse imaging conditions and control experiments on fluorescence noise interference. a) Merged images and FITC fluorescence images from benchtop ddPCR experiments are input SAM‐dPCR for droplet classification testing and droplet diameter measurements. a1. False annotations are removed as indicated by the absence of false rejections. a2. Ablation results without false rejection functions, highlighting the presence of false rejections (highlighted using white arrows in the labeled images and red circles in the plot, respectively). b) Bright‐field images of the same experiment are input SAM‐dPCR for droplet diameter measurement. b1. Workflow of bright‐field image segmentation and plot of droplet diameter measurement. b2. Comparative analysis of droplet diameter measurement results. SAM‐dPCR shows better segmentation and diameter analysis performance when applied to bright‐field images due to less scattering influence compared to the other two imaging modes. c) Dust specks (white arrows) had no detectable impact on segregation accuracy due to their small size relative to microreactors. d) Air bubbles (white arrows) also had no detectable impact on segregation accuracy due to their relatively large size. Scale bar: 100 µm.

The robustness and versatility of SAM‐dPCR were assessed under various imaging conditions, including bright‐field images, fluorescent field images, and merged images that combined fluorescence with bright‐field. Our implementation flags and removes masks with multiple connected components from the sorted list of annotations. **Figure** **4**a1 demonstrates the SAM‐dPCR model's ability to eliminate false annotations, ensuring accurate analysis of microreactor masks. The ablation experiment in Figure 4a2 illustrates false rejections with white arrows and red circles. Figure 4b1 illustrates the workflow for droplet segmentation and diameter measurement using a bright‐field image. Figure 4b2 compares droplet diameter measurements across different imaging modes. The errors for merged field, fluorescence field, and bright‐field are 8.33 µm, 14.96 µm, and 3.89 µm, respectively. This shows SAM‐dPCR's superiority with bright‐field images. Unlike fluorescence imaging, which relies on excitation and emission, bright‐field imaging uses absorption‐based contrast, making it more reliable for precise droplet diameter analysis. Additionally, we systematically adjusted the SNRs by introducing Gaussian noise and salt‐and‐pepper noise, resulting in SNRs range from 9.331 dB to 2.086 dB for ddPCR images (Figure S2, Supporting Information), and 4.979 dB to 1.396 dB for microwell dPCR images (Figure S3, Supporting Information). This capability to distinguish between different signal intensities, even under low SNR conditions, demonstrates the method's sensitivity and precision. While the presence of dark signal droplets indicates potential negatives, the overall superior performance is evidenced by the method's ability to accurately classify droplets across a range of signal intensities. We found that fluorescence noise, such as fouling, dust specks, and air bubbles, had minimal interference with segregation results. We intentionally introduced known amounts of dust (Figure 4c) and air bubbles (Figure 4d), finding no detectable impact on segmentation accuracy. Neither contaminants were detected in the final results. Moreover, we observed negligible spatial variations in background signal and found that the exposure could tolerate a certain degree of light transmission loss. For routine background signal evaluations, SAM‐dPCR employs disposable microfluidic chips.

**Figure 5:**
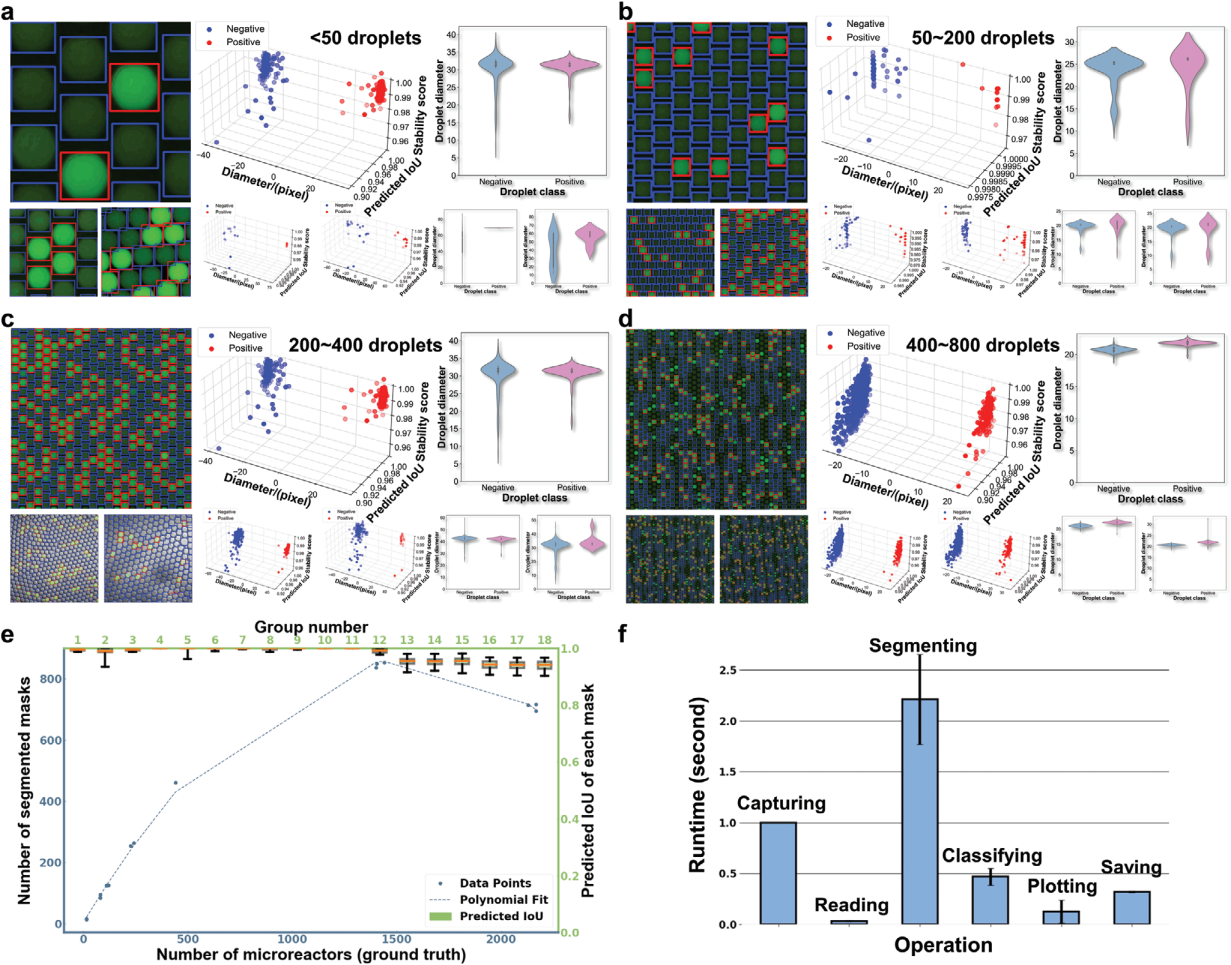
SAM‐dPCR performance across varying droplet counts and runtime testing. a) <50 droplets per image, b) 50–200 droplets per image, c) 200–400 droplets per image, d) 400–800 droplets per image. Each condition includes analysis of three ddPCR images to evaluate SAM‐dPCR robustness and versatility. e) Detection capability analysis by testing dPCR images of microreactor counts per image range from 6 to over 2000. Each dot in the plot represents the results of one dPCR image. The blue plot yields the correlation between detected reactors and actual counts, while the green group illustrates the relationship between Predicted IoU and reactor counts. The comparison between the actual number and the detected number serves to assess the LoD. f) Runtime of the SAM‐dPCR system encompassing the steps of Capturing, Reading, Segmenting, Classifying, Plotting, and Saving. The algorithm demonstrates the capability to simultaneously visualize hundreds of reactors per image in just 3.16 seconds, excluding the capture time.

In addition to imaging conditions, we assessed the LoD by analyzing the detectable droplet count per image and required image size. The mask count, representing detected microreactors, is essential for quantifying target molecules and evaluating image quality. It enables precise sample concentration calculations by calculating positive and negative microreactors. Comparing actual counts with mask counts helps evaluate the method's applicability. The tests in **Figure** **5**a–d involve four conditions with varying droplet counts from an array of dPCR experiments, each varying in sample types, target genes, and experimental conditions. For each condition ranging from < 50 counts to 400–800 counts, three representative dPCR images were analyzed using the SAM‐dPCR model. When the droplet counts fall below 50, significant measurement errors occur due to incomplete microreactors (detailed in Figure S4a, Supporting Information). On the contrary, when the count surpasses 400, miss‐detections increase (Figure S4b, Supporting Information). This limitation likely stems from SAM's training dataset, which contains an average of 100 masks per image.^[^ ^37^ ^]^ This issue can be addressed by fine‐tuning SAM on more specific downstream dPCR image segmentation tasks or cropping images into smaller regions. Figure 5e shows x‐y mappings of detected masks and Predicted IoU, where each image's Predicted IoU is the average of individual reactor Predicted IoUs. 18 dPCR images (12 from Figure 5a–d) were analyzed, with droplet counts per image varying from 6 to 2000. The blue plot shows the correlation between mask counts and true droplet counts. A strong correlation is observed for reactor numbers between 50 and 400, consistent with results in Figure 5a–d. The green group illustrates the relationship between Predicted IoU and mask counts. The 93% Predicted IoU value indicates high accuracy of segmentation predictions, reflecting a strong overlap between predicted masks and ground truth reactors. We tested SAM‐dPCR's practical applicability by applying it under conditions of droplet merging and varying resolutions (from 1024×1024 to 64×64 pixels), as shown in Figure S5 (Supporting Information). The results demonstrate that merged droplets can be correctly segmented and classified. However, resolutions below 256×256 pixels compromise full image detection, as target reactors often diminish to less than 16×16 pixels, impeding feature extraction in dPCR images. Our experiments reveal that 1024×1024 or 512×512 pixels provide the optimal balance of detail and efficiency. A detailed breakdown of SAM‐dPCR's runtime is provided in Figure 5f and Table S3 (Supporting Information). SAM‐dPCR analysis process consists of four primary computational steps: (1) reading images from the fluorescence microscope's application programming interface (API) or software development kit (SDK) cable, requiring approximately 0.03 seconds per image; (2) segmenting and classifying microreactors through SAM‐dPCR, necessitating less than 2.21 and 0.47 seconds per image respectively; (3) plotting and exhibiting the results, takes around 0.13 seconds per image; and (4) outputting the analyzed results and labeled images into a designated folder, taking roughly 0.04 seconds per image. Our image capture setting is configured for one frame per second, which is adjustable. Consequently, our runtime analysis achieves 3.16 seconds including all processing steps beyond the capture phase.

**Figure 6:**
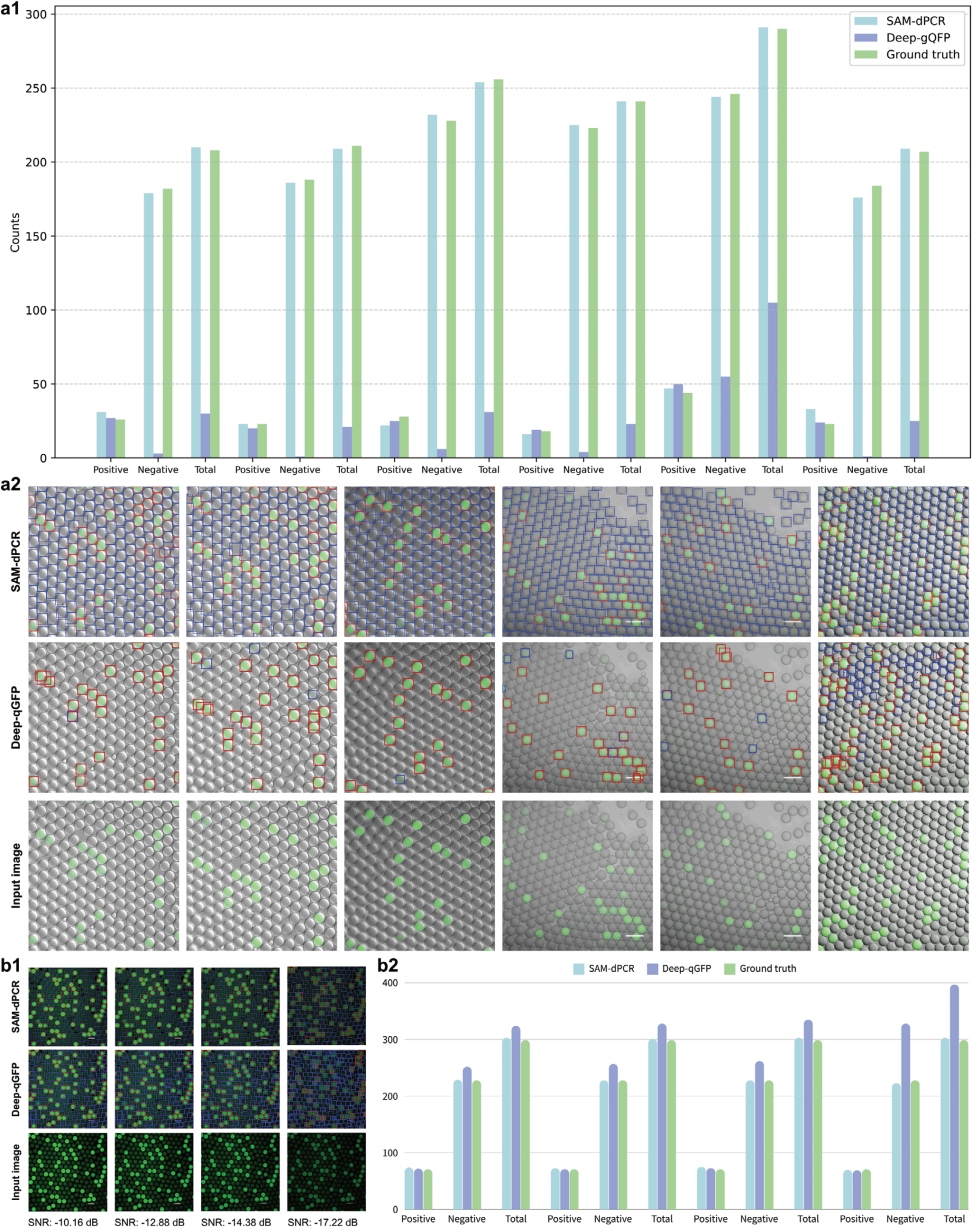
Performance comparison of SAM‐dPCR and fully supervised Deep‐qGFP. a) Performance comparison of SAM‐dPCR and Deep‐qGFP on six ddPCR images (over 1400 droplets in total). a1. Plot showing the results of both models and ground truth. a2. Labeled images demonstrating the segmentation and classification results. SAM‐dPCR achieved an accuracy of 97.10%, higher than Deep‐qGFP's 96.23%. b) Comparative analysis of SAM‐dPCR and Deep‐qGFP on images with different SNRs. SAM‐dPCR had fewer false negatives (0 to 2.19%, or 5 droplets) compared to Deep‐qGFP (10.53% to 43.86%, equivalent to 24 to 100 droplets). This discrepancy for Deep‐qGFP may be due to the training dataset not matching the varied experimental conditions in the testing dataset.

We compared SAM‐dPCR with the fully supervised Deep‐qGFP model (**Figure** **6**). Deep‐qGFP was trained on over 200 manually labeled ddPCR datasets using YOLO‐v5m and the Region Proposal Network (RPN).^[^ ^32^ ^]^ In Figure 6a, we tested six ddPCR images with both models, resulting in analyzing over 1400 droplets (including positive and negative). Figure 6a1 plots the segmentation and classification results with ground truth, while Figure 6a2 presents the labeled images. Detailed experiment records can be found in Tables S4 and S5 (Supporting Information), respectively. SAM‐dPCR achieved an accuracy of 97.10%, higher than Deep‐qGFP's reported 96.23%. The droplet diameter measurement error was 5.177 pixels for SAM‐dPCR and 24.893 pixels for Deep‐qGFP. We also compared the models on different SNR images (Figure 6b). SAM‐dPCR had fewer false negatives (0 to 2.19%, equal to 0 to 5 droplets) compared to Deep‐qGFP (10.53% to 43.86%, equal to 24 to 100 droplets). This result for Deep‐qGFP may be due to the training dataset not matching the varied experimental conditions in the testing dataset. Figure S6 (Supporting Information) further supports these findings across different testing datasets.

**Figure 7:**
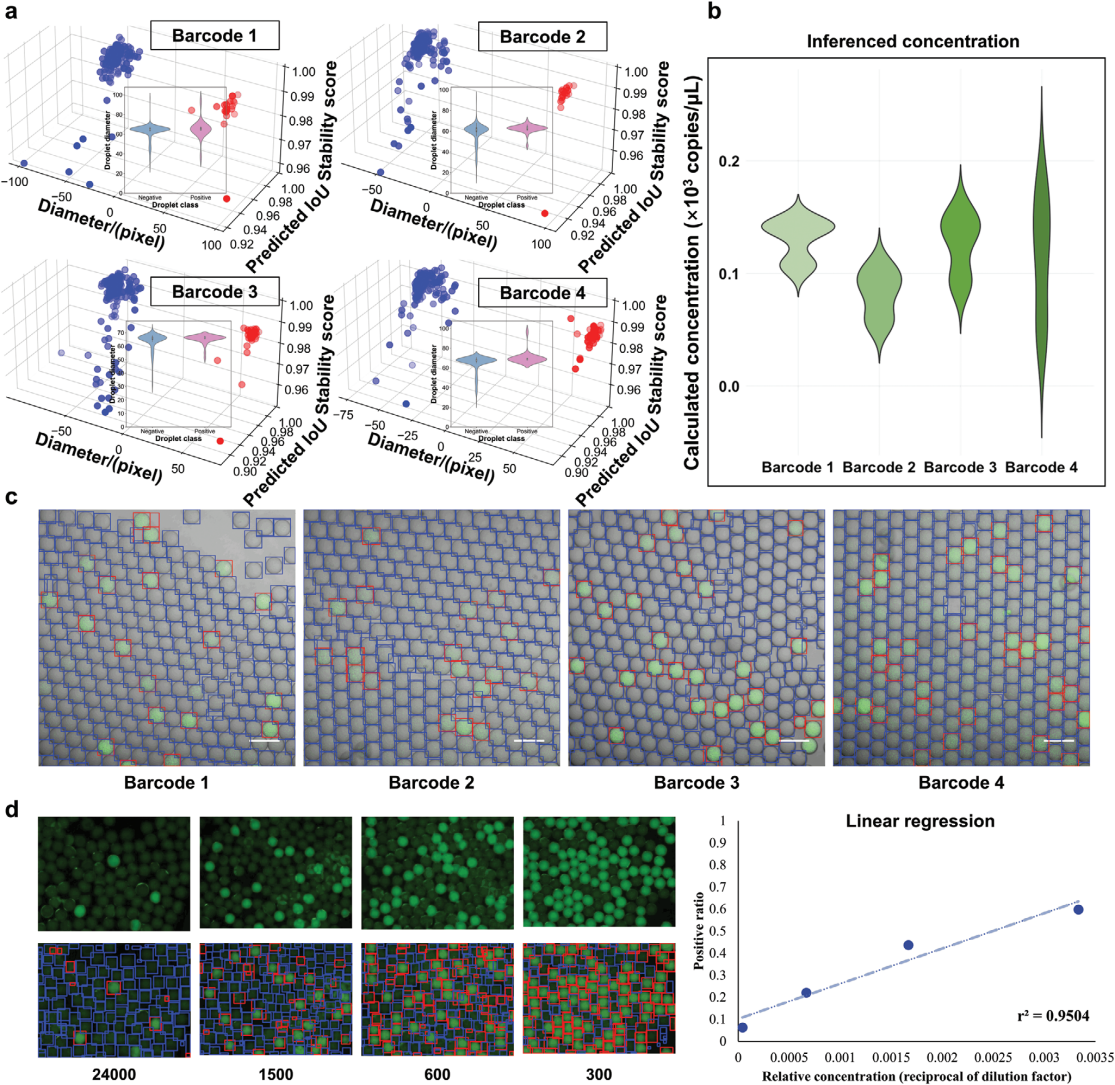
Generalization testing of the SAM‐dPCR model from single‐cell sequencing to hydrogel‐based dPCR. a) Single frame droplet segmentation and classification results for the droplet single‐cell sequencing with varied barcoding. b) Statistical validation of the SAM‐dPCR model's performance, analyzing over 2000 droplets per experimental condition. c) Single‐cell sequencing images in which droplets are labeled using bounding boxes. d) Results of the SAM‐dPCR model applied to hydrogel‐based dPCR images, with 24000, 1500, 600, and 300 times dilution of harvested *S. Typhi* DNA. The linear regression between known concentration and positive ratio (r^2^ = 0.9504) strongly evidences the accuracy and robustness of the model across a wide range of concentrations. Scale bar: 100 µm.

Our SAM‐dPCR exhibits robust generalization capabilities when directly applied to a range of microreactor‐based biological applications, as shown in **Figure** **7**. These microreactors, including droplets, microwells, and hydrogel beads, are visualized through the incorporation of DNA intercalating dyes such as SYBR Green, EvaGreen, Calcein, and Alexa Fluor 488. Our SAM‐dPCR has been validated with varied barcoding (Figure 7a–c), hydrogel‐based dPCR (Figure 7d), and droplet‐based digital quantification of *E. Coli* suspensions (Figure S7, Supporting Information). Specifically, the results for Figure 7a–c has been cross‐validated by comparing with manual counting results in Table S6 (Supporting Information). Figure 7d displays the application of SAM‐dPCR model to hydrogel‐based dPCR images, with serial dilutions of harvested *S. Typhi* DNA at 24 000, 1500, 600, and 300 times. The linear regression between known concentration and positive ratio yields an r^2^ value of 0.9504. In each of these scenarios, the microreactors are automatically and accurately segmented and classified, with the consequent data plotted and concentrations calculated accordingly. This generalizability and versatility across various microreactor‐based applications positions our model favorably in comparison to fully supervised AI models, which typically require extensive training data for each new application.

## 3 Discussion and Conclusion

Digital PCR has undeniably pioneered molecular biology with its precision, sensitivity, and nucleic acid absolute quantification capabilities. Traditional analysis methods, such as manual counting or flow cytometry, however, remain tied to challenges in high costs, complexity, and accessibility limitations. In this study, we present the SAM‐dPCR model for dPCR image analysis that requires no training data, achieving an accuracy of 97.10% with 200∼400 droplets per frame. This model demonstrates superior segmentation capability, high generalizability, and robustness under low SNRs. The novel 3D visualization provides a more detailed and holistic view of the data distribution spatially. By incorporating droplet diameter into the output, readers can readily assess both the class and size of each droplet, enhancing the comprehensibility of our data presentation. It encapsulates wide‐ranging DNA intercalating dye‐labeling scenarios and extends across droplet‐based, microwell‐based, and agarose‐based applications common to biological experiments. It is open‐source and resource‐efficient, reducing the risk of overfitting. Contrasting with commercialized software such as Bio‐Rad's QX Manager which is restricted to Bio‐Rad Droplet Digital PCR Systems, including QX600, QX600 AutoDG, QX200, and QX200 AutoDG, SAM‐dPCR showcases impressive adaptability, negating the need for any specific facilities or trained operators. Additionally, our study introduces offline and real‐time operation modes, showcasing the adaptability of SAM‐dPCR across a variety of dPCR experimental applications and conditions.

Compared to other SOTA models, SAM‐dPCR stands out for its self‐supervised nature, high accuracy, and generalizability (**Table** **1**). Supervised AI‐assisted automatic analysis techniques are limited by heavy reliance on specific training datasets, thereby challenging rapid transformations such as changes in droplet diameters or imaging conditions. For instance, Deep‐qGFP^[^ ^32^ ^]^ requires 206 concentration‐varying ddPCR images training dataset and achieves 96.23% accuracy, showing competitive performance but with higher data dependency. 5‐layer CNN model‐enabled ddPCR classification^[^ ^30^ ^]^ needs 60 ddPCR images, cropped to over 12000 46×46 pixels images, achieving up to 99.71% for single positive droplet classification, indicating very high accuracy for specific tasks but with significant data preprocessing. YOLOv5‐based one‐stage analysis^[^ ^38^ ^]^ uses 40 160 × 160 pixels images, augmented to 120 images, reporting an error rate of 0.65% and a missing rate of 2.17%, showing high accuracy but with generalizability concerns. YOLOv3‐enabled few‐shot detection^[^ ^31^ ^]^ requires 120 training and 40 testing images of chip‐based dPCR and 80 training and 20 testing images of ddPCR, achieving 98.98% accuracy. Mask R‐CNN‐enabled automatic detection^[^ ^39^ ^]^ needs 60 ddPCR images of 640×480 pixels, achieving a true positive ratio of 97.56%, showing high accuracy but with limited adaptability and need for extensive data. Notably, both the high accuracies achieved by YOLOv3 model and Mask R‐CNN were assessed on droplet evenly distributed images with counts less than 130.

**Table 1:** Comparison of SAM‐dPCR and SOTA models.

Name	Model architecture	Required training data volume	Accuracy in dPCR analysis	Generalizability and robustness (especially on low SNRs)	Pros	Cons
SAM‐dPCR (this work) 2024	Self‐supervised: Segment Anything Model	No training data required	Accuracy of 97.10% with 200–400 droplets per frame	Applied on different imaging conditions, different SNRs, different droplet‐based applications, and different fluorescence dyes.	Superior segmentation capability; No training required; High generalizability; Robust segmentation under low SNRs; Open source; Resource efficiency; Reduced overfitting risk.	Challenges with dense object detection (over 600 objects/frame); Model complexity;
Deep‐qGFP^[^ ^32^ ^]^ 2023	Supervised: YOLOv5 with RPN	206 concentration‐varying ddPCR images	Accuracy of 96.23% with ∼200 droplets within one frame	Applied on different imaging conditions. Demonstrated ddPCR, microwell dPCR, agarose dPCR, and other droplet‐based applications.	High quantification abilities; High adaptability to specific droplet qGFP scenarios.	Miss‐detection for small objects; Higher data requirements; Potential for overfitting.
CNNs‐enabled ddPCR classification^[^ ^30^ ^]^ 2023	Supervised: Hough transform and CNNs	60 ddPCR images of 2456 × 2404 pixels, cropped to 12000+ 46×46 pixels images	Up to 99.71% for single positive droplet classification	Tested on variable exposure, contrast, and uneven illumination conditions.	Automation; Potential high speed after being optimized.	Parameter tuning is required; High data requirement for training.
YOLOv5‐based one‐stage analysis^[^ ^38^ ^]^ 2023	Supervised: YOLOv5 with CBAM	40 160 × 160 pixels images, augmented to 120 images.	Error rate 0.65%, missing rate 2.17% with <180 droplets per image.	The dataset was prepared by reducing the brightness or applying Gaussian noise.	Simplified process; Accessible via mobile devices or cloud platforms.	Generalizability concern; Resource intensive.
YOLOv3‐enabled few‐shot detection^[^ ^31^ ^]^ 2021	Supervised: YOLOv3, Random Background Transfer Method (RBTM), and Source Traceability Annotation Method (STAM)	120 training and 40 testing images of chip‐based dPCR; 80 training and 20 testing images of ddPCR. Resolution: 1600×1200 pixels	98.98% with <130 droplets per image. TP:2735; FP:39; TN:3531; FN:1;	Applied on both chip‐based dPCR and ddPCR images. Tested on internal irregular noise and uneven illumination datasets.	Effective for limited data (augment images and reduce the labeling time by 70%); Better optimal accuracy and processing speed than YOLOv3 and Mask R‐CNN	Potential lower accuracy; Dependency on initial data.
Mask R‐CNN‐enabled automatic detection^[^ ^39^ ^]^ 2019	Supervised: Mask R‐CNN with GMM	60 ddPCR images of 640×480 pixels.	A true positive ratio of 97.56% with ≤121 droplets per image.	Tested on uneven light images and impure images.	Labeling time‐cost reduced; Streamlined detection process; Potential for high throughput.	Limited adaptability; Need for extensive data.

We calculated expected concentrations and compared them with inferred concentrations in Table S7 (Supporting Information). We acknowledge discrepancies between these values, which can be attributed to several factors: the tested concentrations may not reach the expected maximum, indicating limitations in the assay's dynamic range, potential DNA adsorption to tube surfaces, or PCR inhibitors like the oil phase in droplet dPCR; contamination with extraneous DNA or degradation over time could lead to lower than expected copy numbers, especially at higher concentrations; and variability in dPCR instrument performance or reagent quality could contribute to observed discrepancies. To address these issues, future studies will optimize sample preparation, dilution procedures, and PCR efficiency.

The difference between droplet and microwell formats mainly stems from their partition mechanisms and reactor uniformity. Droplet dPCR partitions samples into thousands of water‐in‐oil emulsions, each acting as an individual reaction chamber, offering high sensitivity but susceptible to droplet variability and PCR efficiency issues due to the oil phase. Microwell dPCR partitions samples into thousands of individual microwells, typically on a microfluidic chip or plate. This method demonstrates better reactor uniformity and robustness against sample contamination but may exhibit lower sensitivity. To improve accuracy within this range, we incorporated a correction factor, $P_{r}\left(X=2\right)$, for concentrations where λ > 1. Additionally, we conducted supplementary experiments that demonstrated the method's capability to detect sample concentrations as low as 0.154 copies µL^−1^ (Figure S8, Supporting Information). These findings, along with the correction factor, offer a solid foundation for investigating the method's potential applicability at even lower concentrations in future research.

Our SAM‐ddPCR model's performance is contingent on the quality of dPCR images, necessitating clear and sharp visuals for precise microreactor segmentation and classification. To further enhance the results, we optimized the imaging settings, from focusing and exposure conditions to magnifications (ranging from 4 × to 20×), droplet sizes (diameter between 18 and 114 pixels), and not disregarding image resolutions (ranging from 256×256 to 4140×3096 pixels), applied to both bright‐field and fluorescence field images. In this work, we selected SAM for its superior adaptability in diverse imaging scenarios, eliminating the need for extensive retraining or fine‐tuning. SAM can operate with three encoders – ViT‐B, ViT‐L, and ViT‐H,^[^ ^37^ ^]^ each with differing parameter counts. Our comparisons, highlighted in Figure S9 (Supporting Information), demonstrated that while there is a negligible difference in droplet segmentation and measurement between the ViT‐B and ViT‐H encoders, ViT‐B offers notably higher classification accuracy. Furthermore, ViT‐H is more susceptible to errors from fluorescence intensity sensitivity and duplication, as evidenced in the provided images. With the substantial parameter count leap from ViT‐B's 91 million to ViT‐H's 636 million, we prioritized ViT‐B for SAM‐dPCR. This choice enables swifter processing and maintains model simplicity and accessibility, crucial for laboratory adoption.

In conclusion, our results underscore the generalist, high‐throughput capacity of SAM‐dPCR for accurate and dependable quantification of biological samples. Pioneering the employment of a self‐supervised AI model, SAM‐dPCR overcomes the need for pristine or “ground truth” data, a feat made challenging by the inherently unique nature of each amplification process. This adaptability endorses SAM‐dPCR as an advantageous tool for researchers’ intent on astute and efficient quantification of biological samples within disparate experimental frameworks. Looking ahead, the open‐source method, SAM‐dPCR, holds the potential for modification to cater to multiplex tests in dPCR. This could be achieved through crafting fluorescent probes with different emission spectra that specifically target varying molecules or organizing fluorescence intensity to differentiate between targets^[^ ^33^, ^35^, ^40^, ^41^ ^]^. Additionally, we see an avenue of further improvement in exploring stain‐less image analysis or virtual histological staining of biological samples for label‐free^[^ ^35^, ^42^, ^43^, ^44^ ^]^ and non‐invasive diagnosis.^[^ ^45^ ^]^ This progress could simplify the technical demands for fluorescent dyes and sophisticated optics for detection. Furthermore, recent advancements in nucleic acid sensors^[^ ^46^ ^]^ and signal amplification biosensors^[^ ^47^ ^]^ present additional opportunities for the application of SAM‐dPCR in the development of advanced sensing technologies for investigating cell membrane penetrability and nuclease degradation. These advances, which include enhanced in vivo imaging and diagnostic capabilities, address critical challenges such as tissue penetration, cell membrane penetrability, and nuclease degradation. Integrating these strategies into SAM‐dPCR could significantly improve its detection sensitivity, specificity, and clinical applicability, thereby expanding its potential in diverse biomedical applications.

## 4 Experimental Section

### Sample Preparation

High‐fidelity Q5 DNA Polymerase (New England Biolabs) was employed as the primary commercial kit for the ddPCR reactions. The ddPCR reaction mix comprised diluted restriction enzymes, dNTPs, buffer, forward and reverse primers, Tween‐20, PEG‐8000, and PCR water. The detailed composition of the ddPCR reagents were outlined in Table S8 (Supporting Information). The cDNA templates were serially diluted for the ddPCR experiments, maintaining a stock cDNA concentration of (1.12 ± 0.09) × 10^4^ copies µL^−1^.

For the ddPCR experiments depicted in Figure 2, the PCR mixture was formulated with 1X KAPA HIFI buffer, 0.3 mM dNTP, 1X KAPA HIFI polymerase, 0.3 µM forward and reverse primers, templates, 0.1% NP‐40, 0.2% Tween 20, and 0.1 mg mL^−1^ BSA (NEB, USA). Template concentrations of 40 pg, 4 pg, and 0.4 pg per 20 µL PCR system were used. The PCR protocol was initiated with denaturation at 95 °C for 3 min, followed by 45 cycles of denaturation at 98 °C for 20 s, annealing at 61 °C for 15 s, and extension at 72 °C for 15s. The final extension was conducted at 72 °C for 1 min, with an indefinite hold at 12 °C. The stability of the samples was ensured throughout the ddPCR process and during storage at 4 °C for 48 hours. Measurement of droplets after the PCR thermal amplification process can be found in Figure S10d (Supporting Information).

The extracted Seahorse (Hippocampus kuda) genome, targeting the *cytochrome c oxidase subunit I (COI)* with an amplicon size of 206 bp, was utilized as the PCR template. The forward and reverse primer sequences were as follows. The design has been validated in the previous work^[^ ^48^ ^]^.

Forward primer (5→3): TTTCTTCTCCTCCTTGCTTCCTCAG

Reverse primer (5→3): GAAATTGATGGGGGTTTTATGTTG

For the ddPCR experiments in Figure 4, the PCR mixture incorporated 1X Platinum SuperFi II buffer, 0.2 mM dNTP, 1X Platinum SuperFi II polymerase, 0.5 µM forward and reverse primers, templates, 0.2% Tween 20, 0.2 mg mL^−1^ BSA (NEB, USA), and 0.4% PEG‐8000. The PCR protocol included initial denaturation at 98 °C for 30 s, followed by 45 cycles of denaturation at 98 °C for 10 s, annealing at 60 °C for 10 s, and extension at 72 °C for 15s. The final extension was performed at 72 °C for 5 min with an indefinite hold at 12 °C. The sequences of the templates and primers used were detailed as follows:

The PCR template (5→3):

GTCTCGTGGAGCTCGACAGCATNNNNNNTGNNNNNNTGCCTACGACAAACAGACCTAAAATCGCTCATTGCATACTCTTCAATCAG

Forward primer (5→3):

Acrydite‐ACTAACAATAAGCTCUAUAGTCTCGTGGAGCTCGACAG

Reverse primer (5→3):

CTGATTGAAGAGTATGCAATGAG

In the single‐cell sequencing experiments (Figure 6), the PCR mixture was prepared using 1X TaKaRa PrimeSTAR GXL buffer, 0.2 mM dNTP, 1X TaKaRa PrimeSTAR GXL polymerase, 0.2 µM forward and reverse primers, templates, 0.5% Tween 20, 0.1 mg mL^−1^ BSA (NEB, USA), and 0.5% PEG‐8000. The PCR program was initiated with 25 cycles at 95 °C for 10 s, 59 °C for 15 s, and 68 °C for 15 s, followed by a final extension at 68 °C for 3 min and a hold at 25 °C. The sequences of templates and primers are provided in **Table** **2**.

**Table 2:** Sequences of templates and primers in single‐cell sequencing experiments.

			Sequence (5→3)
Barcode 1	Template		GTCTCGTGGAGCTCGACAGNNNNNNNNNNNN TCGCTCATTGCATACTCTTCAATCAGC
	Primer	Forward	GTCTCGTGGAGCTCGACAG
		Reverse	GCTGATTGAAGAGTATGCAATG
Barcode 2	Template		GTCTCGTGAGTCAGGACAGNNNNNNNNNNNN TCGCTCATTGCATACTCTTCAATCAGC
	Primer	Forward	GTCTCGTGAGTCAGGACAG
		Reverse	GCTGATTGAAGAGTATGCAATG
Barcode 3	Template		GTCTCGTGACCTCGGACAGNNNNNNNNNNNN TCGCTCATTGCATACTCTTCAATCAGC
	Primer	Forward	GTCTCGTGACCTCGGACAG
		Reverse	GCTGATTGAAGAGTATGCAATG
Barcode 4	Template		GTCTCGTGGACAGTGACAGNNNNNNNNNNNN TCGCTCATTGCATACTCTTCAATCAGC
	Primer	Forward	GTCTCGTGGACAGTGACAG
		Reverse	GCTGATTGAAGAGTATGCAATG

### Microfluidic Chip Fabrication and Droplet Generation

A flow‐focusing microfluidic chip with cross‐sectional dimensions of 30.0 µm (width) and 38.5 µm (height) was designed and fabricated for droplet generation (Figure S10a–c, Supporting Information). The chip was created using standard SU‐8 photolithography and Polydimethylsiloxane (PDMS) replica molding processes. Initially, a master mold of 60.0 µm height was prepared by spinning SU8‐2075 photoresist at 5000 r.p.m. for 28 s on a 4″ silicon wafer, followed by UV exposure, baking, and developer bath according to the manufacturer's specifications. PDMS prepolymers (Dow, Inc., Sylgard 184) with a base‐to‐curing ratio of 10:1 were degassed, poured onto the SU‐8 masters, and baked at 60 °C for 10 h. The PDMS was then removed and cut to the desired shape, with inlets and outlets punched through. Both the PDMS slabs and glass slides were treated with oxygen plasma for 1 min and bonded by brief baking at 110 °C. Finally, the microfluidic devices were hydrophobized by baking at 55 °C for 24 h.

Monodisperse emulsions were generated using a custom microfluidic setup, driven by two syringe pumps (Legato 100, KD Scientific or Ph.D. 2000, Harvard Apparatus, USA) at the inlets. Commercial droplet generation oil (Bio‐Rad Laboratories, Inc.) was used as the continuous phase. The resulting droplets exhibited a uniform size distribution with a mean diameter of 46.37 ± 1.64 µm (0.052 nL). The generated droplets were collected and transferred into 0.2 mL PCR stripe tubes, which were covered with mineral oil to minimize evaporation. PCR reactions were performed in a thermal cycler (Bio‐Rad) with a hot start at 94 °C for 5 min, followed by 55 cycles of denaturation at 94 °C for 30 s, annealing, and extension at 60 °C for 1 min. Subsequently, the droplets were isolated onto a glass slide for observation.

### Microreactors Imaging and Image Processing

The amplified droplets were collected and dispensed into a specially designed PDMS chamber for observation under a lab fluorescence microscope. Imaging of the emulsions was performed using an inverted microscope (Eclipse Ti‐U, Nikon) coupled with a camera (DS‐Qi2, Nikon) in both brightfield and fluorescence fields. Fluorescence excitation was achieved at 455 nm, and the emitted light was captured by a CCD through a 495 nm long‐pass filter. Images were acquired at 4 × magnification with an exposure time of 1 s and a sensor sensitivity of 200. For each sample, 10 images were taken at different positions, excluding edges, encompassing over 2000 droplets in total. The droplet diameter was quantified and analyzed using *ImageJ* (National Institutes of Health and the Laboratory for Optical and Computational Instrumentation).

The microwell dPCR images presented in this manuscript were acquired using a 3D Digital PCR chip v2, with each reaction well having a volume of 755 pL (ThermoFisher Scientific, USA). These experiments were conducted and validated by this collaborator, Prof. Mingli You, from the School of Life Science and Technology at Xi'an Jiaotong University.^[^ ^33^ ^]^ The experimental design involved a series of dilutions, spanning a broad dynamic range of template concentrations, i.e., negative, 100×, 20×, 10 × and 5 × dilutions from the original concentration of 1.66 × 10^−13^ mol L^−1^. This study amplified two types of double‐stranded genes, specifically blaNDM and blaVIM, which regulate the expression of β‐lactamases, a class of antibiotic agents used against carbapenems. The corresponding DNA sequences can be accessed on the GenBank website (https://www.ncbi.nlm.nih.gov/genbank/) using the nucleotide codes NC023908 (137 552 bp) and NC023274 (38 403 bp) for blaNDM and blaVIM, respectively. Due to the extensive length of the complete gene sequences, specific subsequences were selected as templates and subsequently redesigned the corresponding primers and probes. These genes were synthesized as plasmids by QingsKe, based in Xi'an, China, who also synthesized all primers. The probes for blaNDM and blaVIM, labeled as blaNDM‐AF488 and blaVIM‐TET respectively, were modified with AF488 and TET, and were synthesized by Sangon, based in Shanghai, China.

The hydrogel‐based dPCR images in Figure 7d were extracted from publication with permission.^[^ ^49^ ^]^ The original images in Figure S7 (Supporting Information) were extracted from previously published bacterial quantification work.^[^ ^50^ ^]^

### Deep‐Learning‐Assisted Automatic Data Analysis

The SAM‐dPCR algorithm was based on the zero‐shot SAM architecture, which produces high‐quality masks without requiring training. The SAM model utilizes a CNN architecture with multiple layers to extract robust features through convolutions, pooling, and normalization operations. Trained on a dataset of 11 million images and 1.1 billion masks (dataset SA‐1B, available at https://segment‐anything.com), SAM enhances and extracts relevant features from input images. The dataset categorizes images based on the number of masks per image, with an average of around 100 masks per image. These masks were generated using automated segmentation techniques and a semi‐supervised approach, resulting in high‐quality and diverse masks validated through human ratings and extensive experiments.

After mask generation, the algorithm converts images to the HSV color space, utilizes Otsu's method for auto‐thresholding, and categorizes microreactors as “positive” or “negative” according to mean fluorescence intensity. Each microreactor was assessed based on diameter, Predicted IoU, and Stability Score, derived from segmentation consistency within a single image. To calculate the Stability Score, the model slightly alters the dPCR image (e.g., pixel values or feature maps) and compares the segmentation masks from the original and adjusted images. The similarity between these masks determines the Stability Score. This fully automated process does not necessitate any further user input beyond the initial image.

The entire process was executed using the Pytorch deep learning framework on an NVIDIA Tesla V100‐SXM2‐16GB hardware platform. Detailed instructions for the construction and operation of SAM‐dPCR can be found at https://github.com/WEI‐yuanyuan/SAM‐dPCR.

The probability P_r_(*X* = *k*) that a microreactor will contain *k* copies of target gene if the mean number of target copies per microreactor is λ:

(1) $$f\left(k,\lambda \right)=P_{r}\left(X=k\right)=\frac{\lambda^{k}e^{-\lambda }}{k!}$$

where
*k* is the number of occurrences (*k* can take values 0, 1, 2, …).
*e* is Euler's number (*e* = 2.71828…).
! is the factorial function.

Inputting *k* = 0 gives the probability that a microreactor will be empty:

(2) $$P_{r}\left(X=0\right)=e^{-\lambda }$$

For the number of microreactors being large enough, the observed fraction of empty microreactors (E) gives the estimation of P_r_(*X* = *k*)

(3) $$E=e^{-\lambda }$$

At the same time, by definition of E,

(4) $$E=\frac{N_{negative}}{N}$$

Solving (3),

(5) $$\lambda =-\ln\left(E\right)$$

As λ is the copies per microreactor, the concentration of copies per volume is

(6) $$Concentration=\frac{\lambda }{V_{microreactor}}$$

Here mathematical corrections were applied for high template concentrations (λ > 1) by using a correction factor *P_r_* (*X* = 2):

(7) $$\lambda^{\prime }=\lambda +2\times P_{r}\left(X=2\right)$$

λ′ was the corrected average number of target molecules per partition, considering partitions that may contain more than one template,

Thus for concentrations with λ > 1, their respective concentrations were corrected to:

(8) $$Concentration=\frac{\lambda^{\prime }}{V_{microreactor}}$$

To ensure transparency, the detailed calculations and results of these probabilities for each experimental setup are included in Tables S1 and S2 (Supporting Information).

The accuracy of SAM‐dPCR was calculated based on Equation 9. The calculation process can be found in Table S9 (Supporting Information).

(9) $$ACC=\frac{TP+TN}{TP+TN+FP+FN}$$

### Graphical User Interface

SAM‐dPCR algorithm was implemented as a standalone software tool using Python and packaged as a user‐friendly GUI. The GUI allowed users to interact with the software, input dPCR images, and visualize the segmentation results in real‐time. The software was designed to seamlessly integrate with common laboratory fluorescence microscopes, enabling easy adoption and utilization in different experimental setups. The GUI also plots the droplets by accumulating results from sequential frames. Users have the option to digitally save the raw images, background‐subtracted images, plot results, and calculated values. The size of the droplets and the calculated template concentration were continuously displayed at the bottom. Additionally, the GUI can operate in offline mode to analyze pre‐saved image datasets by reading folders. The design codes for the GUI can be found in the Supporting Materials.

### Statistical Analysis

Statistical analysis was performed using GraphPad Prism software (GraphPad Software). All data were presented as mean ± standard deviation (SD) with n ≥ 3. Hypothesis testing was conducted using a t‐test, and significance was defined as *p* ≤ 0.05.

The r^2^ value in this work represents the coefficient of determination. It was computed using the standard formula for linear regression:

(10) $$r^{2}=1-\frac{\sum {\left(y_{i}-{\hat{y}}_{i}\right)}^{2}}{\sum {\left(y_{i}-{\bar{y}}_{i}\right)}^{2}}$$

where
*y_i_* was the observed value,
${\hat{y}}_{i}$ was the predicted value from the regression model, and
${\bar{y}}_{i}$ was the mean of the observed data.

This formula quantifies the proportion of the variance in the dependent variable that was explained by the independent variable(s) in the model.

## Conflict of Interest

The authors declare no conflict of interest.

## Author Contributions

Y.W., S.L., and W.Y. contributed to the study's conception and design, with S.L. specifically realizing the software functions and implementation. Y.W. and F.Q. conducted the biological experiments. Y.W., S.L., C.X., and Y.F. performed image analysis experiments, data analysis, and figure editing. F.Q. and Y.‐P.H. supplied the microfluidic chip and customized microfluidic platform. G.Z. revised the manuscript and gave valuable comments. Y.W. wrote the manuscript with contributions from all authors. W.Y. and H.‐P.H. conceived the project and supervised the research.

## Supporting information



Supporting Information

## Data Availability

The data that support the findings of this study are available from the corresponding author upon reasonable request.
