# Supplementary material for: SAM‐dPCR: Accurate and Generalist Nuclei Acid Quantification Leveraging the Zero‐Shot Segment Anything Model
Source: Adv Sci (Weinh). 2024 Dec 27;12(7):2406797. doi: 10.1002/advs.202406797 (PMC11831435; doi:10.1002/advs.202406797)
Supplement: Supplementary file 1 — Supporting Information [file ADVS-12-2406797-s001.docx]

**Supporting Information**

# SAM-dPCR: Accurate and Generalist Nuclei Acid Quantification Leveraging the Zero-Shot Segment Anything Model

*Yuanyuan Wei^1^, Shanhang Luo^2^, Changran Xu^3^, Yingqi Fu^1^, Yi Zhang^4^, Fuyang Qu^1^, Guoxun Zhang^5^, Yi-Ping Ho^1, 6, 7, 8^, Ho-Pui Ho^1, *^, Wu Yuan^1, *^*

^1^ Department of Biomedical Engineering, The Chinese University of Hong Kong, Shatin, Hong Kong SAR, 999077, China. E-mail: [aaron.ho@cuhk.edu.hk](mailto:aaron.ho@cuhk.edu.hk); [wyuan@cuhk.edu.hk](mailto:wyuan@cuhk.edu.hk)

^2^ Department of Biomedical Engineering, National University of Singapore, 119077, Singapore

^3^ Department of Computer Science and Engineering, The Chinese University of Hong Kong, Shatin, Hong Kong SAR, 999077, China

^4^ Department of Electronic Engineering, The Chinese University of Hong Kong, Shatin, Hong Kong SAR, 999077, China

^5^ Department of Automation, Tsinghua University, Beijing, 100084, China

^6^ Centre for Biomaterials, The Chinese University of Hong Kong, Hong Kong SAR, 999077, China

^7^ Hong Kong Branch of CAS Center for Excellence in Animal Evolution and Genetics, Hong Kong SAR, 999077, China

^8^ State Key Laboratory of Marine Pollution, City University of Hong Kong, Hong Kong SAR, 999077, China

| **Supplementary Table S1** | Statistical analysis records of microwell dPCR experiments. |
| --- | --- |
| **Supplementary Table S2** | Statistical analysis records of droplet dPCR experiments. |
| **Supplementary Table S3** | Runtime calculation record of SAM-dPCR. |
| **Supplementary Table S4** | Supplement table for Figure 6a: Performance comparison of SAMdPCR and Dee-qGFP across ddPCR images. |
| **Supplementary Table S5** | Supplement table for Figure 6b: Performance comparison of SAM-dPCR and Deep-qGFP across different SNR levels. |
| **Supplementary Table S6** | Comparison of SAM-dPCR model and manual counting results for different barcods. |
| **Supplementary Table S7** | Comparison between expected concentration and inferred concentration. |
| **Supplementary Table S8** | ddPCR reagents for a 25 µL premix. |
| **Supplementary Table S9** | SAM-dPCR accuracy calculation. |

| **Supplementary Figure 1** | SAM-dPCR GUI enables real-time and high-throughput analysis of microreactors. |
| --- | --- |
| **Supplementary Figure 2** | Depiction of SAM-qPCR's segmentation efficacy in the face of significant noise challenges. |
| **Supplementary Figure 3** | Representative images portraying segmentation performance within microwell dPCR across a range of SNRs. |
| **Supplementary Figure 4** | Limitations observed in SAM-dPCR analysis results. |
| **Supplementary Figure 5** | Practical applicability of SAM-dPCR under varying conditions. |
| **Supplementary Figure 6** | Performance evaluation of SAM-dPCR compared to the fully supervised Deep-qGFP model. |
| **Supplementary Figure 7** | Comparative assessment of SAM-dPCR with ViT-B and ViT-H encoders. |
| **Supplementary Figure 8** | SAM-dPCR demonstration on low sample concentration. |
| **Supplementary Figure 9** | Results of droplet-based digital bacterial quantification under varying suspension conditions. |
| **Supplementary Figure 10** | Microfluidic chip design for uniform droplet generation and characterization. |

**Supplementary Table S1. Statistical analysis records of microwell dPCR experiments**.

The microwell dPCR images presented in **Figure 3** were acquired using a 3D Digital PCR chip v2, with each reaction well having a volume of 755 pL (ThermoFisher Scientific, USA). The inferred concentration of each sample is calculated as below.

| Sample concentration | Frame | Pos | Neg | Total | Sum Pos | Sum Neg | Sum  Total | λ = -  ln(Sum  _Neg/  Sum_T otal) | Pr(0) | Pr(1) | Pr(2) | Pr(≥  1) |
| --- | --- | --- | --- | --- | --- | --- | --- | --- | --- | --- | --- | --- |
| 1.66 × 10^−15^ mol/L | 1 | 28 | 179 | 207 | 231 | 1801 | 2032 | 0.121 | 0.886 | 0.107 | 0.007 | 0.114 |
|  | 2 | 26 | 178 | 204 |  |  |  |  |  |  |  |  |
|  | 3 | 19 | 204 | 223 |  |  |  |  |  |  |  |  |
|  | 4 | 20 | 195 | 215 |  |  |  |  |  |  |  |  |
|  | 5 | 25 | 198 | 223 |  |  |  |  |  |  |  |  |
|  | 6 | 31 | 187 | 218 |  |  |  |  |  |  |  |  |
|  | 7 | 29 | 180 | 209 |  |  |  |  |  |  |  |  |
|  | 8 | 18 | 157 | 175 |  |  |  |  |  |  |  |  |
|  | 9 | 13 | 163 | 176 |  |  |  |  |  |  |  |  |
|  | 10 | 22 | 160 | 182 |  |  |  |  |  |  |  |  |
|  | Inferred concentration | | | | 0.160 × 10^3^ copies/µL | | | | | | | |
| Sample concentration | Frame | Pos | Neg | Total | Sum Pos | Sum Neg | Sum  Total | λ = -  ln(Sum  _Neg/  Sum_T otal) | Pr(0) | Pr(1) | Pr(2) | Pr(≥  1) |
| 8.30 × 10^−15^ mol/L | 1 | 92 | 125 | 217 | 822 | 1203 | 2025 | 0.521 | 0.594 | 0.309 | 0.081 | 0.406 |
|  | 2 | 84 | 127 | 211 |  |  |  |  |  |  |  |  |
|  | 3 | 74 | 136 | 210 |  |  |  |  |  |  |  |  |
|  | 4 | 92 | 131 | 223 |  |  |  |  |  |  |  |  |
|  | 5 | 85 | 126 | 211 |  |  |  |  |  |  |  |  |
|  | 6 | 78 | 141 | 219 |  |  |  |  |  |  |  |  |
|  | 7 | 89 | 121 | 210 |  |  |  |  |  |  |  |  |
|  | 8 | 74 | 102 | 176 |  |  |  |  |  |  |  |  |
|  | 9 | 75 | 100 | 175 |  |  |  |  |  |  |  |  |
|  | 10 | 79 | 94 | 173 |  |  |  |  |  |  |  |  |
|  | Inferred concentration | | | | 0.690 × 10^3^ copies/µL | | | | | | | |
| Sample concentration | Frame | Pos | Neg | Total | Sum Pos | Sum Neg | Sum  Total | λ = -  ln(Sum  _Neg/  Sum_T otal) | Pr(0) | Pr(1) | Pr(2) | Pr(≥  1) |
| 1.66 × 10^−14^ mol/L | 1 | 149 | 69 | 218 | 1316 | 701 | 2017 | 1.057 | 0.348 | 0.367 | 0.194 | 0.652 |
|  | 2 | 126 | 83 | 209 |  |  |  |  |  |  |  |  |
|  | 3 | 141 | 74 | 215 |  |  |  |  |  |  |  |  |
|  | 4 | 136 | 74 | 210 |  |  |  |  |  |  |  |  |
|  | 5 | 136 | 83 | 219 |  |  |  |  |  |  |  |  |
|  | 6 | 132 | 73 | 205 |  |  |  |  |  |  |  |  |
|  | 7 | 129 | 85 | 214 |  |  |  |  |  |  |  |  |
|  | 8 | 130 | 45 | 175 |  |  |  |  |  |  |  |  |
|  | 9 | 123 | 53 | 176 |  |  |  |  |  |  |  |  |
|  | 10 | 114 | 62 | 176 |  |  |  |  |  |  |  |  |
|  | Inferred concentration before correction | | | |  |  | 1.400 × 10^3^ copies/µL | | | |  |  |
|  | Expected number of molecules per microwell  λ^′^ =λ+2⨯𝛲_𝑟_(𝛸 =2) | | | |  |  | 1.445 | | | |  |  |
|  | Inferred concentration after correction | | | |  |  | 1.914 × 10^3^ copies/µL | | | |  |  |
| Sample concentration | Frame | Pos | Neg | Total | Sum Pos | Sum Neg | Sum  Total | λ = -  ln(Sum  _Neg/  Sum_T otal) | Pr(0) | Pr(1) | Pr(2) | Pr(≥  1) |
| 3.32 × 10^−14^ mol/L | 1 | 198 | 22 | 220 | 1811 | 225 | 2036 | 2.203 | 0.111 | 0.243 | 0.268 | 0.889 |
|  | 2 | 190 | 21 | 211 |  |  |  |  |  |  |  |  |
|  | 3 | 189 | 21 | 210 |  |  |  |  |  |  |  |  |
|  | 4 | 182 | 29 | 211 |  |  |  |  |  |  |  |  |
|  | 5 | 192 | 26 | 218 |  |  |  |  |  |  |  |  |
|  | 6 | 191 | 21 | 212 |  |  |  |  |  |  |  |  |
|  | 7 | 200 | 21 | 221 |  |  |  |  |  |  |  |  |
|  | 8 | 155 | 21 | 176 |  |  |  |  |  |  |  |  |
|  | 9 | 159 | 22 | 181 |  |  |  |  |  |  |  |  |
|  | 10 | 155 | 21 | 176 |  |  |  |  |  |  |  |  |
|  | Inferred concentration before correction | | | |  |  | 2.918 × 10^3^ copies/µL | | | |  |  |
|  | Expected number of molecules per microwell  λ^′^ =λ+2⨯𝛲_𝑟_(𝛸 =2) | | | |  |  | 2.739 | | | |  |  |
|  | Inferred concentration after correction | | | |  |  | 3.629 × 10^3^ copies/µL | | | |  |  |
|  | r^2^ without correction | | | |  |  | 0.9992 | | | |  |  |
|  | r^2^ with correction | | | |  |  | 0.9935 | | | |  |  |

**Supplementary Table S2. Statistical analysis records of droplet dPCR experiments**.

Similarly, for droplet dPCR results in **Figure 2**, the droplet diameter is 85.29 ± 4.35 µm (equivalent to a volume of 325.00 pL). The inferred concentration of each sample is as below.

| Sample concentration | Frame | Pos | Neg | Total | Sum Pos | Sum Neg | Sum  Total | λ = -  ln(Su m_Ne g/  Sum_  Total) | Pr(0) | Pr(1) | Pr(2) | Pr(≥1) |
| --- | --- | --- | --- | --- | --- | --- | --- | --- | --- | --- | --- | --- |
| 0.02pg/µL | 1 | 14 | 290 | 304 | 48 | 1101 | 1149 | 0.043 | 0.958 | 0.041 | 0.001 | 0.042 |
|  | 2 | 18 | 282 | 300 |  |  |  |  |  |  |  |  |
|  | 3 | 8 | 274 | 282 |  |  |  |  |  |  |  |  |
|  | 4 | 16 | 272 | 288 |  |  |  |  |  |  |  |  |
|  | 5 | 6 | 273 | 279 |  |  |  |  |  |  |  |  |
|  | Inferred concentration | | | |  |  | 0.132 × 10^3^ copies/µL | | | |  |  |
| Sample concentration | Frame | Pos | Neg | Total | Sum Pos | Sum Neg | Sum  Total | λ = -  ln(Su m_Ne g/  Sum_  Total) | Pr(0) | Pr(1) | Pr(2) | Pr(≥1) |
| 0.20pg/µL | 1 | 14 | 186 | 200 | 171 | 1072 | 1243 | 0.148 | 0.862 | 0.128 | 0.009 | 0.138 |
|  | 2 | 27 | 184 | 211 |  |  |  |  |  |  |  |  |
|  | 3 | 31 | 195 | 226 |  |  |  |  |  |  |  |  |
|  | 4 | 34 | 178 | 212 |  |  |  |  |  |  |  |  |
|  | 5 | 14 | 174 | 188 |  |  |  |  |  |  |  |  |
|  | 6 | 51 | 155 | 206 |  |  |  |  |  |  |  |  |
|  | Inferred concentration | | | |  |  | 0.455 × 10^3^ copies/µL | | | |  |  |
| Sample concentration | Frame | Pos | Neg | Total | Sum Pos | Sum Neg | Sum  Total | λ = -  ln(Su m_Ne g/  Sum_  Total) | Pr(0) | Pr(1) | Pr(2) | Pr(≥1) |
| 2.00pg/µL | 1 | 84 | 193 | 277 | 578 | 1105 | 1683 | 0.421 | 0.656 | 0.276 | 0.058 | 0.344 |
|  | 2 | 86 | 148 | 234 |  |  |  |  |  |  |  |  |
|  | 3 | 79 | 179 | 258 |  |  |  |  |  |  |  |  |
|  | 4 | 82 | 159 | 241 |  |  |  |  |  |  |  |  |
|  | 5 | 86 | 133 | 219 |  |  |  |  |  |  |  |  |
|  | 6 | 76 | 145 | 221 |  |  |  |  |  |  |  |  |
|  | 7 | 85 | 148 | 233 |  |  |  |  |  |  |  |  |
|  | Inferred concentration | | | |  |  | 1.295 × 10^3^ copies/µL | | | |  |  |
| r^2^ | | | | |  |  | 0.9643 | | | |  |  |

**Supplementary Table S3. Runtime calculation record of SAM-dPCR**.

| Number | Capturing/s | Reading/s | Segmenting/s | Classifying/s | Plotting/s | Saving/s | Total /s |
| --- | --- | --- | --- | --- | --- | --- | --- |
| 1 | 1 | 0.033 | 2.95 | 0.44 | 0.33 | 0.33 |  |
| 2 | 1 | 0.042 | 2.30 | 0.62 | 0.08 | 0.33 |  |
| 3 | 1 | 0.033 | 1.95 | 0.43 | 0.08 | 0.32 |  |
| 4 | 1 | 0.027 | 1.94 | 0.43 | 0.08 | 0.31 |  |
| 5 | 1 | 0.025 | 1.92 | 0.43 | 0.08 | 0.31 |  |
| Average |  | 0.032 | 2.21 | 0.47 | 0.13 | 0.32 | 3.16 |

**Supplementary Table S4. Supplement table for Figure 6a: Performance comparison of SAMdPCR and Dee-qGFP across ddPCR images.**

| Input image | | Figure 1 | Figure2 | Figure3 | Figure4 | Figure 5 | Figure6 |
| --- | --- | --- | --- | --- | --- | --- | --- |
| SAM-dPCR | Positive | 31 | 23 | 22 | 16 | 47 | 33 |
|  | Negative | 179 | 186 | 232 | 225 | 244 | 176 |
|  | Total | 210 | 209 | 254 | 241 | 291 | 209 |
| Deep-qGFP | Positive | 27 | 20 | 25 | 19 | 50 | 24 |
|  | Negative | 3 | 1 | 6 | 4 | 55 | 1 |
|  | Total | 30 | 21 | 31 | 23 | 105 | 25 |
| Ground truth | Positive | 26 | 23 | 28 | 18 | 44 | 23 |
|  | Negative | 182 | 188 | 228 | 223 | 246 | 184 |
|  | Total | 208 | 211 | 256 | 241 | 290 | 207 |

**Supplementary Table S5. Supplement table for Figure 6b: Performance comparison of SAM-dPCR and Deep-qGFP across different SNR levels.**

| Input image | | Figure 1 | Figure2 | Figure3 | Figure4 |
| --- | --- | --- | --- | --- | --- |
| SNR (dB) | | -10.16 | -12.88 | -14.38 | -17.22 |
| SAM-dPCR | Positive | 74 | 73 | 75 | 70 |
|  | Negative | 229 | 228 | 228 | 223 |
|  | Total | 303 | 301 | 303 | 303 |
| Deep-qGFP | Positive | 72 | 71 | 73 | 69 |
|  | Negative | 252 | 257 | 262 | 328 |
|  | Total | 324 | 328 | 335 | 397 |
| Groundtruth | Positive |  |  | 71 |  |
|  | Negative | 228 | | | |
|  | Total | 299 | | | |

**Supplementary Table S6.** Comparison of SAM-dPCR model and manual counting results for different barcods.

| Barcode | Image |  | SAM-dPCR model | | |  | Ground truth (manual counting) | | |
| --- | --- | --- | --- | --- | --- | --- | --- | --- | --- |
|  |  | Calculated concentration  (× 10^3^ copies µL^-1^) | Positive count | Positive count error | Negative count | Negative count error | Positive count | Negative count | Sum |
| 1 | 1 | 0.142 | 38 | 5 | 385 | 4 | 43 | 389 | 432 |
|  | 2 | 0.142 | 27 | 1 | 239 | 0 | 28 | 239 | 267 |
|  | 3 | 0.107 | 20 | -1 | 237 | 4 | 19 | 241 | 260 |
| 2 | 1 | 0.103 | 20 | -1 | 247 | 1 | 19 | 248 | 267 |
|  | 2 | 0.086 | 17 | 5 | 252 | -11 | 22 | 241 | 263 |
|  | 3 | 0.057 | 19 | 3 | 524 | 10 | 22 | 534 | 556 |
| 3 | 1 | 0.135 | 16 | -1 | 149 | -6 | 15 | 143 | 158 |
|  | 2 | 0.150 | 36 | 3 | 496 | 3 | 39 | 499 | 538 |
|  | 3 | 0.093 | 30 | 0 | 250 | -17 | 30 | 233 | 263 |
| 4 | 1 | 0.124 | 14 | -1 | 143 | -6 | 13 | 137 | 150 |
|  | 2 | 0.050 | 17 | 11 | 520 | -8 | 28 | 512 | 540 |
|  | 3 | 0.170 | 34 | -1 | 249 | 1 | 33 | 250 | 283 |

We calculated the expected concentrations of each dPCR experiment for further comparison.

For a double-stranded DNA fragment, the molecular weight per base pair is approximately 650 daltons.

Therefore, the molecular weight for the 206 bp fragment in ddPCR is:

daltons

𝑀𝑜𝑙𝑒𝑐𝑢𝑙𝑎𝑟 𝑤𝑒𝑖𝑔ℎ𝑡 = 206 bp × 650 = 133900 daltons (1) bp

Since the Avogadro's number 𝑁_𝐴_ is 6.022×10^23^ molecules/mol, the conversion factor from daltons to grams is 1 gram/mol=1×10^12^ pg/mol, the number of molecules per picogram is

6.022 × 10_23_ molecules

𝑀𝑜𝑙𝑒𝑐𝑢𝑙𝑒𝑠/𝑝𝑔 = ^mol^ = 4.49 × 10^5^molecules/pg (2)

daltons pg

133900 × 1 × 1012

mol mol

Thus, the expected concentration for 0.4 pg DNA template per 20 µL PCR system is:


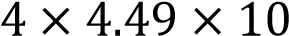


𝐸𝑥𝑝𝑒𝑐𝑡𝑒𝑑 𝑐𝑜𝑛𝑐𝑒𝑛𝑡𝑟𝑎𝑡𝑖𝑜𝑛 = ^0. 5^ = 8.98 × 10^3^𝑐𝑜𝑝𝑖𝑒𝑠/µ𝐿 (3)


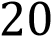


Similarly, we calculated the expected concentrations for microwell dPCR as below:

**Supplementary Table S7. Comparison between expected concentration and inferred concentration**.

|  |  | droplet |  |  | microwell | |  |
| --- | --- | --- | --- | --- | --- | --- | --- |
| Template concentration | 0.2 pg/µL | 2 pg/µL | 20 pg/µL | 1.66 ×  10−15 mol/L | 8.30 ×  10−15 mol/L | 1.66 ×  10−14 mol/L | 3.32 ×  10−14 mol/L |
| Expected concentration  (× 10^3^ copies/µL) | 8.98 | 89.80 | 898.0 | 1.000 | 4.998 | 9.996 | 19.990 |
| Inferred concentration  (× 10^3^ copies/µL) | 0.132 | 0.455 | 1.295 | 0.160 | 0.690 | 1.914 | 3.629 |

**Supplementary Table S8. ddPCR reagents for a 25 µL premix**.

| Reagent |  | Volume/μL |
| --- | --- | --- |
| Reaction Mix | 5x buffer | 5 |
|  | dNTP | 0.5 |
| DNA polymerase |  | 0.25 |
| Primers | F3 | 1 |
|  | B3 | 1 |
| Tween 20 |  | 1 |
| PEG 8000 |  | 1 |
| Template |  | 2 |
| PCR H_2_O |  | 13.25 |
| Total |  | 25 |

**Supplementary Table S9. SAM-dPCR accuracy calculation.**

| Input image | TP | TN | FP | FN |
| --- | --- | --- | --- | --- |
| 1 | 26 | 177 | 5 | 2 |
| 2 | 19 | 184 | 4 | 1 |
| 3 | 22 | 227 | 0 | 5 |
| 4 | 15 | 222 | 1 | 3 |
| 5 | 44 | 244 | 3 | 0 |
| 6 | 21 | 172 | 12 | 5 |
| 𝑇𝑃 + 𝑇𝑁  𝐴𝐶𝐶 = = 97.10%  𝑇𝑃 + 𝑇𝑁 + 𝐹𝑃 + 𝐹𝑁 | | | | |

# Supplementary Figures


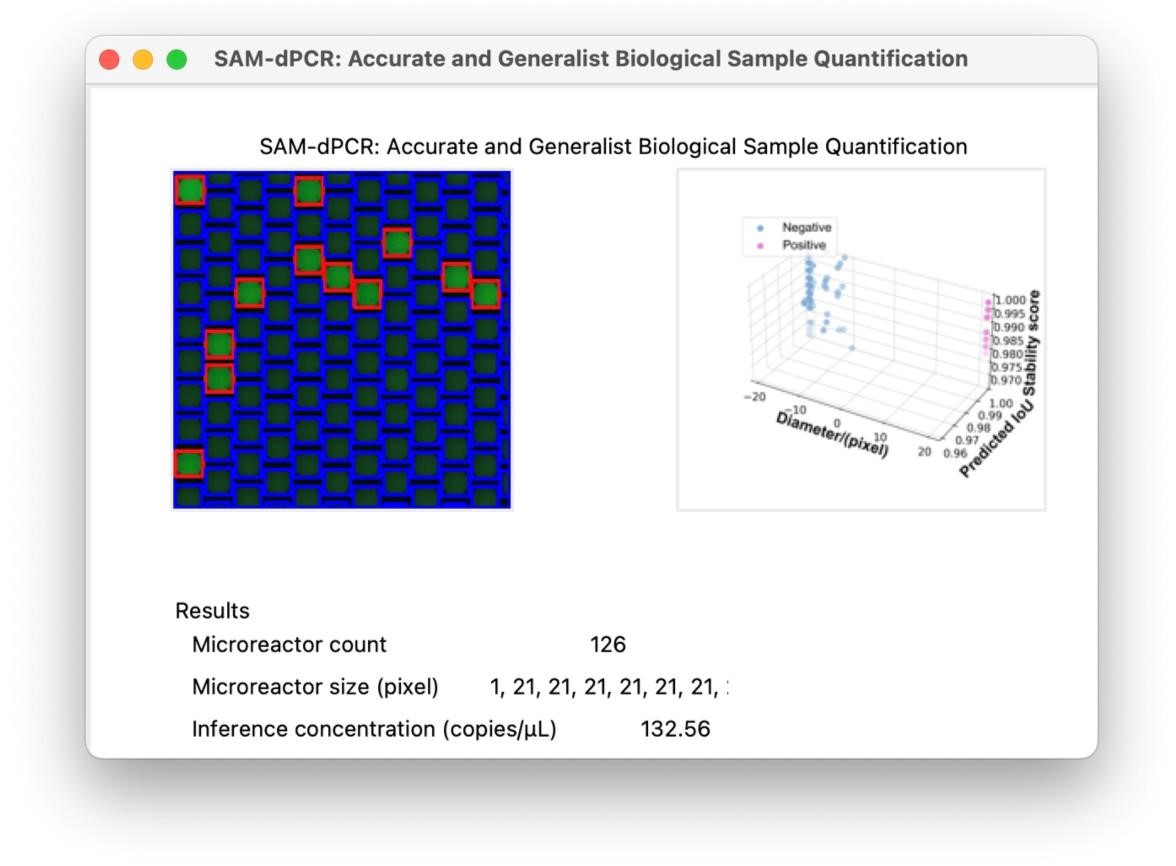


**Figure S1. SAM-dPCR GUI enables real-time and high-throughput analysis of microreactors.** In real-time mode, the GUI displays captured images and analysis results simultaneously. The plotted results are obtained by aggregating frames captured at a rate of one frame per second, achieved by moving the sample stage of the fluorescence microscope. Inferred concentration relies on positive and negative fluorescence data from sample droplets, along with data fitting to a Poisson distribution. Additionally, the GUI supports an offline mode that reads pre-saved images from folders.


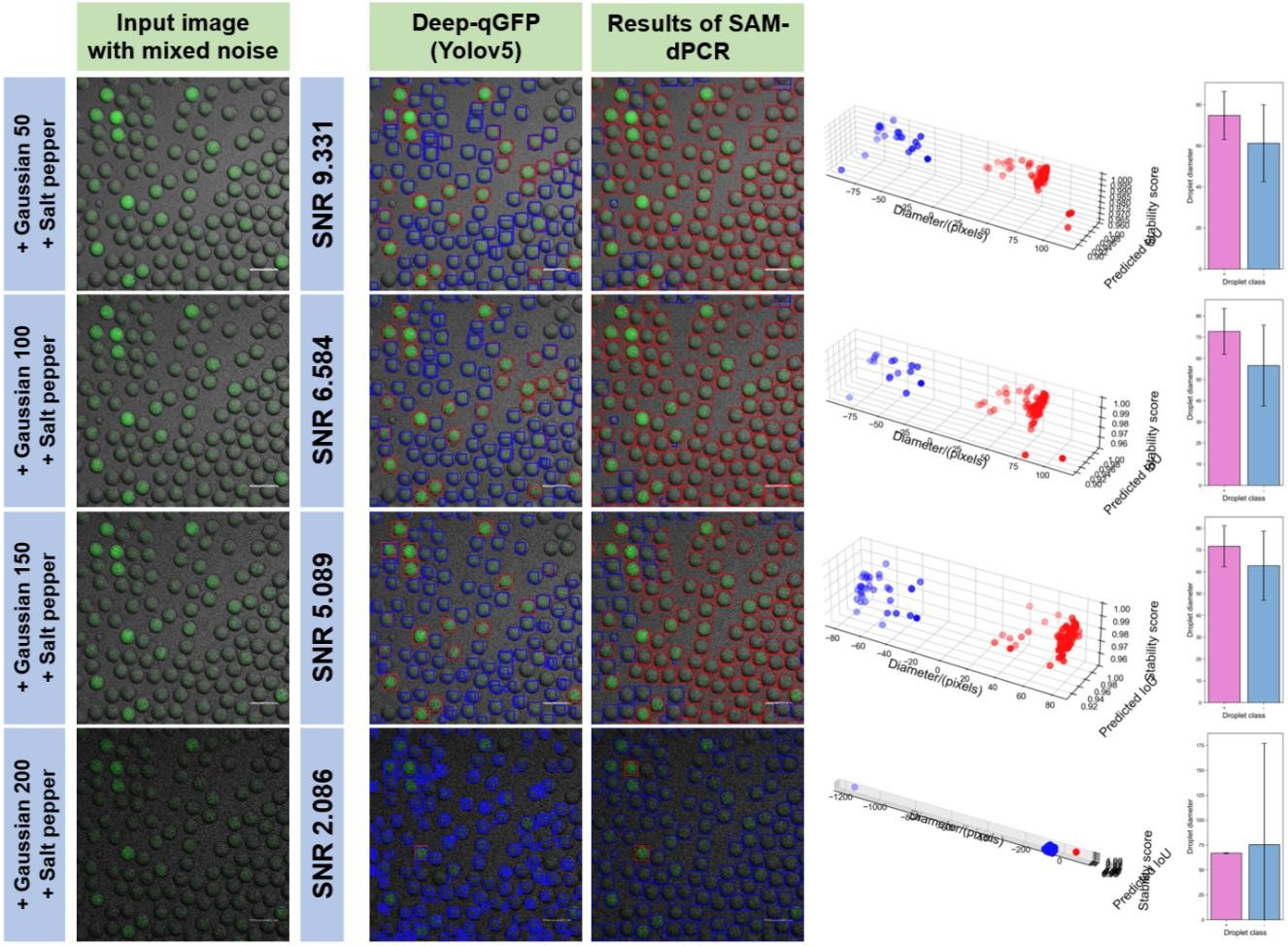


**Figure S2. Depiction of SAM-qPCR's segmentation efficacy in the face of significant noise challenges.** Through a series of segmentation trials that spanned signal-to-noise ratios (SNRs) from 9.331 dB to 2.086 dB, this figure elucidates the comparative resilience and superior segmentation precision of SAM-dPCR over the Yolov5-based Deep-qGFP algorithm, especially notable in complex scenarios involving dense target detection and invalid droplet exclusion under variable SNRs and lighting conditions. The images vividly demonstrate the contrasting degradation patterns, affirming SAM-qPCR's exceptional adaptability and accuracy in less-thanideal imaging environments.


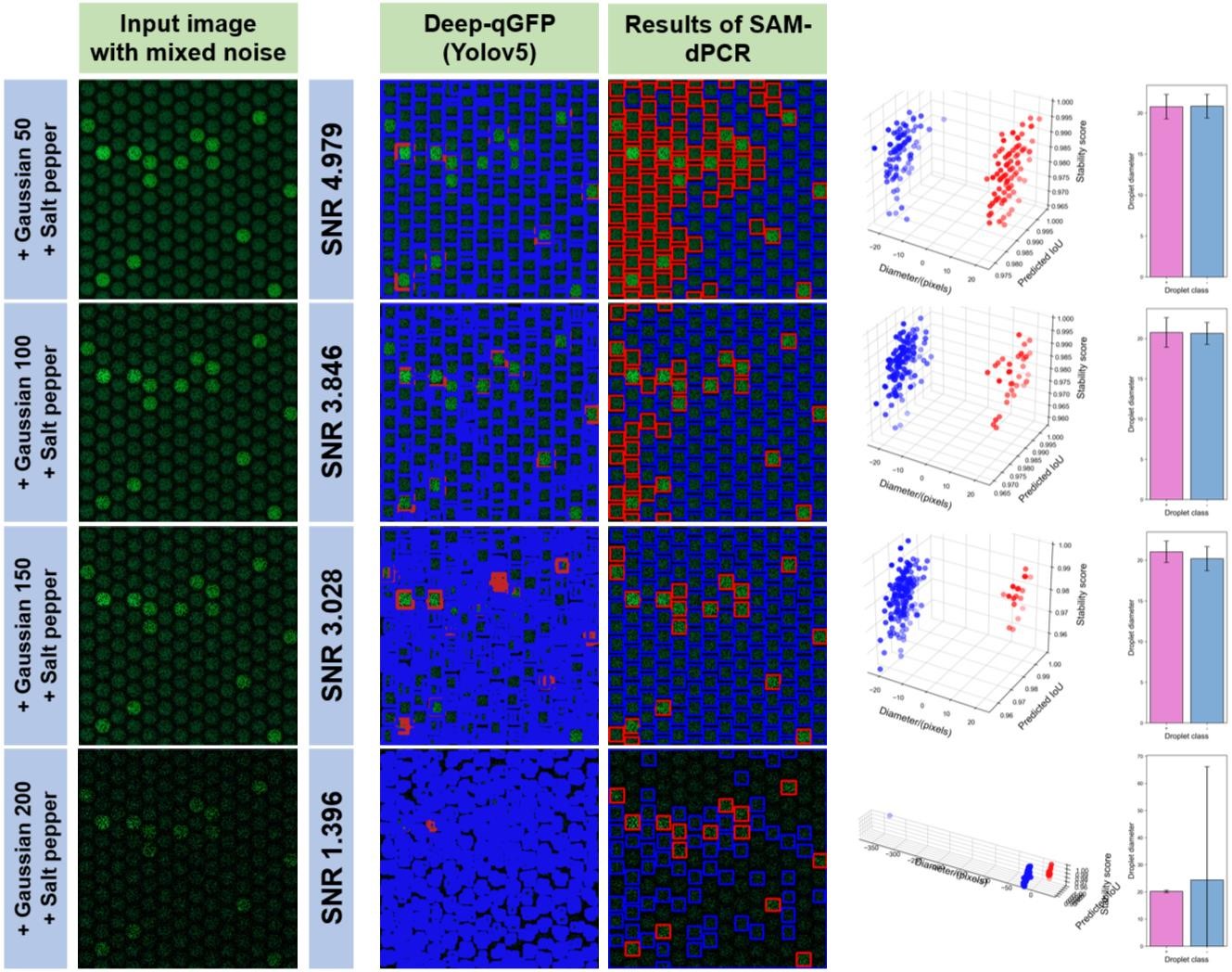


**Figure S3. Representative images portraying segmentation performance within microwell dPCR across a range of SNRs.** This figure presents a meticulous evaluation of SAM-dPCR's segmentation capacity under varying SNRs, benchmarked from 4.979 dB to a modest 1.396 dB. It highlights the algorithm's resilience and superior accuracy in distinguishing densely packed targets and eliminating invalid wells despite the complexities of fluctuating SNRs and lighting in the microwell dPCR setup.


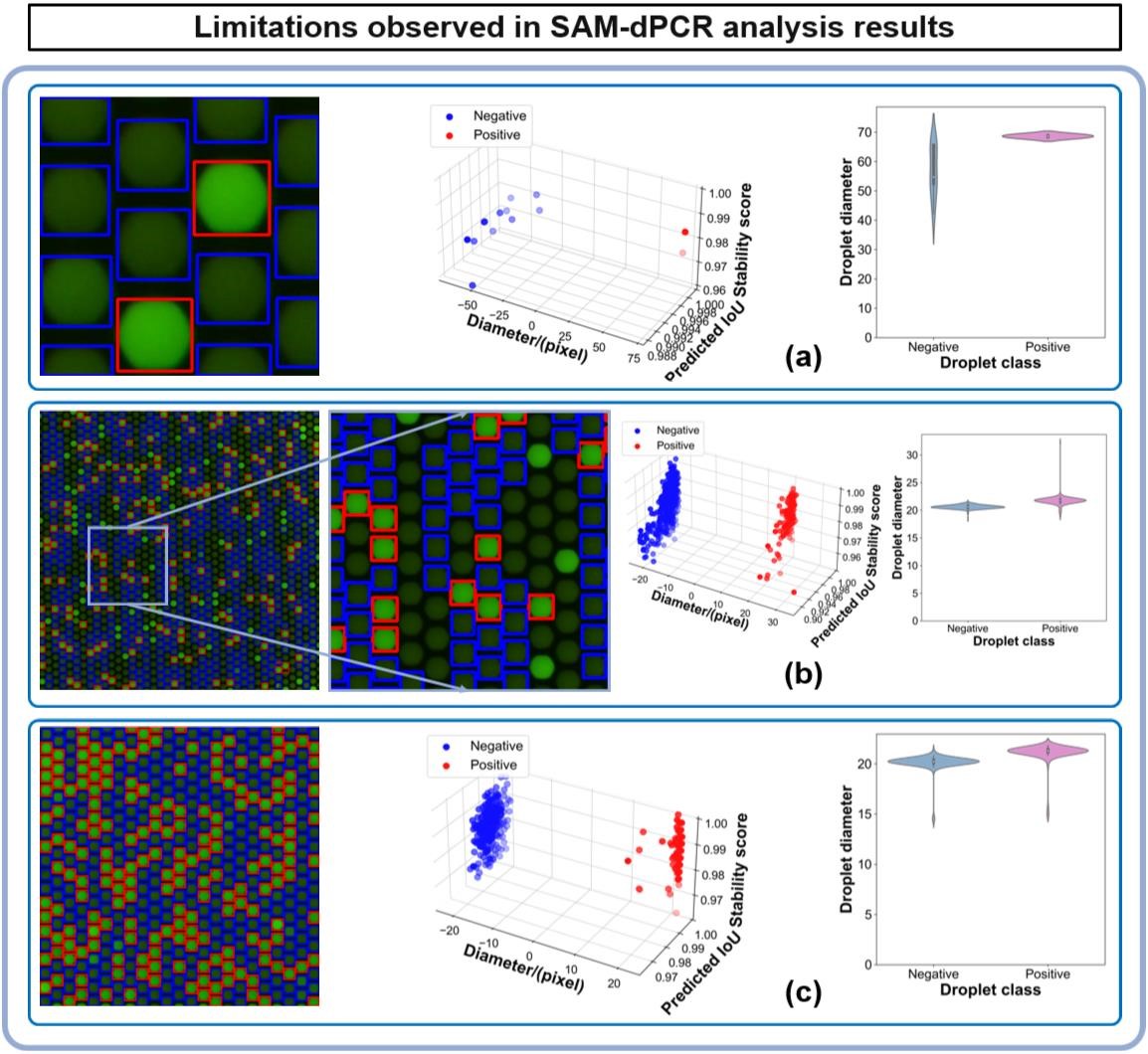


**Figure S4. Limitations observed in SAM-dPCR analysis results.** (a) Measurement errors in reactor size are prominent when the number of masks is less than 100, due to incomplete microreactors in the testing image. (b) SAM-dPCR fails to accurately segment and classify droplets when the number of masks exceeds 600. This limitation arises from the training dataset of SAM, which contains an average of approximately 100 masks per image. This issue can be solved by fine-tuning the SAM model on more specific downstream dPCR image segmentation tasks or cropping the image into smaller regions with fewer than 400 microreactors.


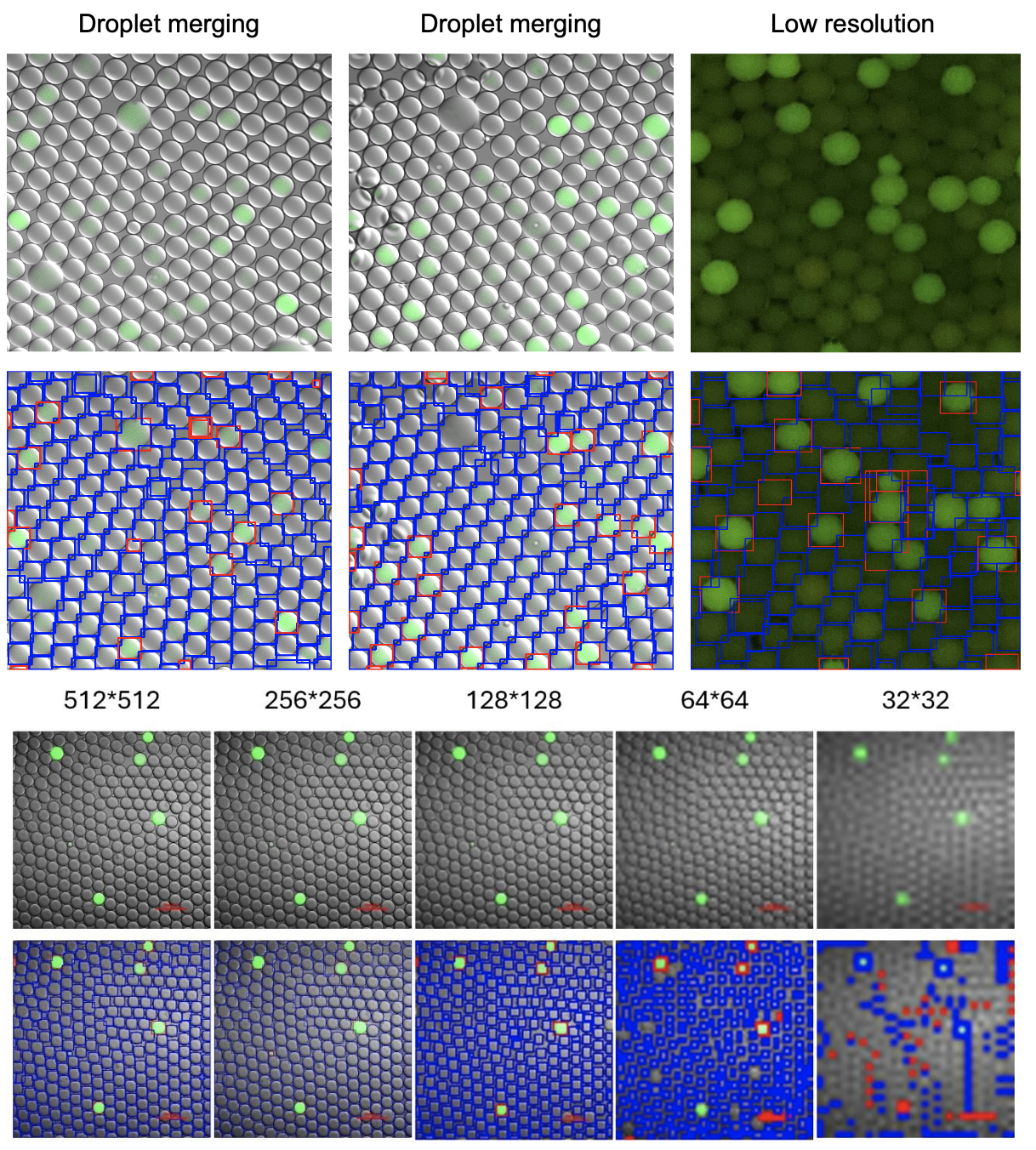


**Figure S5.** **Practical applicability of SAM-dPCR under varying conditions.** The SAM-dPCR demonstrates the ability to correctly segment and classify merged droplets. For lower image resolutions results indicate that sizes below 256×256 pixels compromise feature recognition due to small target sizes (<16×16 pixels). The optimal initial sizes for our dataset are determined to be 1024×1024 or 512×512 pixels, offering a balance between image detail and computational efficiency.


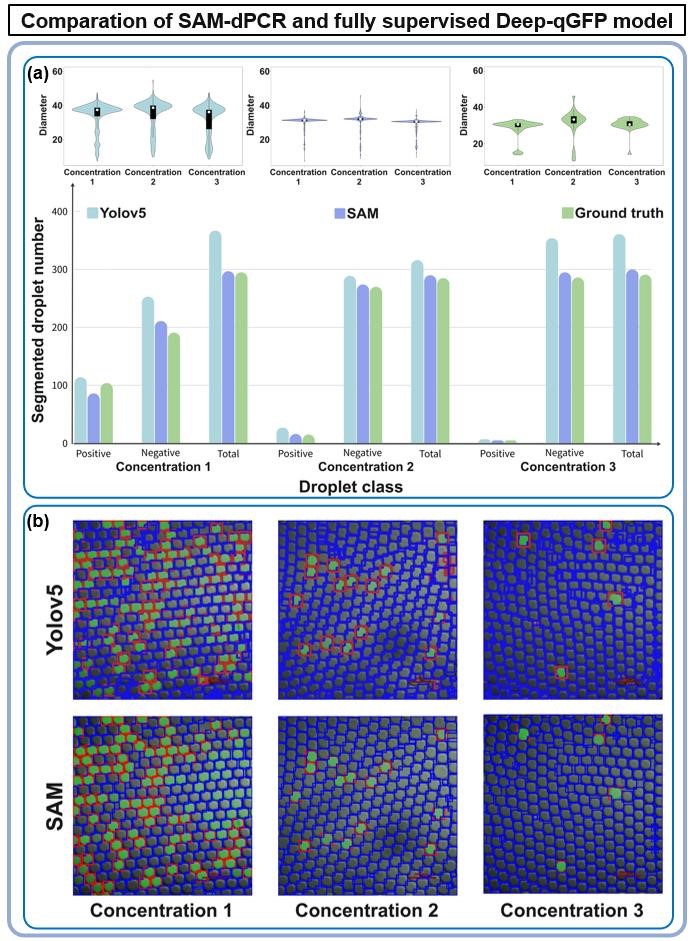


**Figure S6. Performance evaluation of SAM-dPCR compared to the fully supervised DeepqGFP model.** (a) SAM-dPCR accurately estimates droplet diameter with an average deviation of 1.370 pixels, while Deep-qGFP consistently underestimates droplet diameter with an average deviation of 3.551 pixels. This discrepancy can be attributed to non-uniform fluorescence distribution in the testing dataset due to varied experimental conditions. (b) SAM-dPCR achieves a higher accuracy of 97.178% in droplet number counting compared to Deep-qGFP's 96.23%.

SAM-dPCR also exhibits a lower positive droplet count error of 2.201% (equivalent to 6.333 droplets), while Deep-qGFP has an error of 2.565% (equivalent to 8 droplets). Notably, SAMdPCR competes with or even surpasses the fully supervised Deep-qGFP results, despite not requiring a manually annotated training dataset. Manual counting and ImageJ measurement serve as the ground truth for comparison Scale bar: 100 μm.


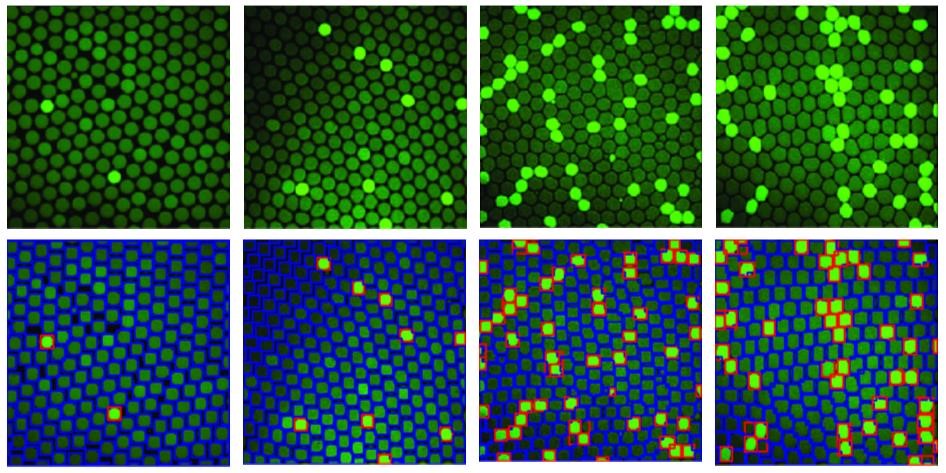


**Figure S7. Results of droplet-based digital bacterial quantification under varying suspension conditions.** Dilutions of E. coli corresponding to theoretical droplet occupancies ranging from 1.7 to 29% are analyzed using the SAM-dPCR model. The labeled images further demonstrates its analytical capability with the dye Alexa Fluor 488.


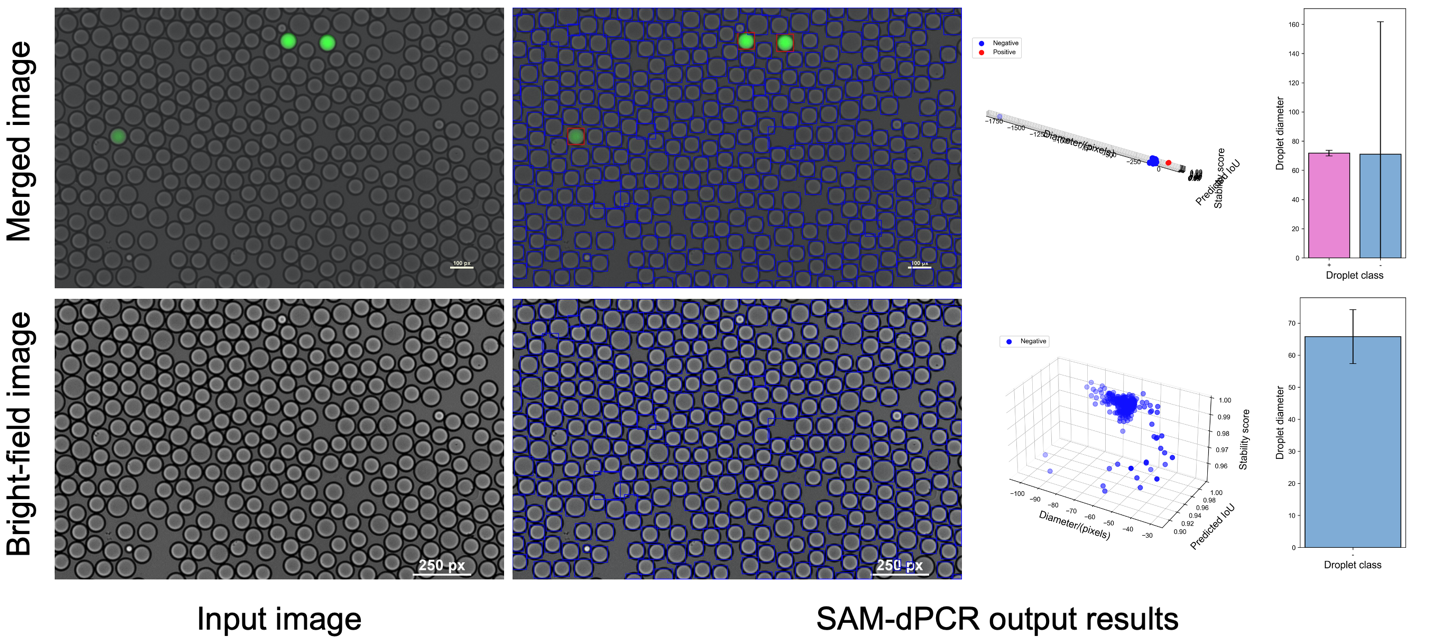


**Figure S8. SAM-dPCR demonstration on low sample concentration.** By applying SAM-dPCR on bright-field image, the droplet diameter was measured to be 69.54 ± 11.79 μm (equivalent to the volume of 176.08 pL). From merged image, positive droplet was counted to be 3 and negative counted to be 330. The λ was calculated to be 0.009. Thus the sample concentration was determined as 0.154 copies/μL.


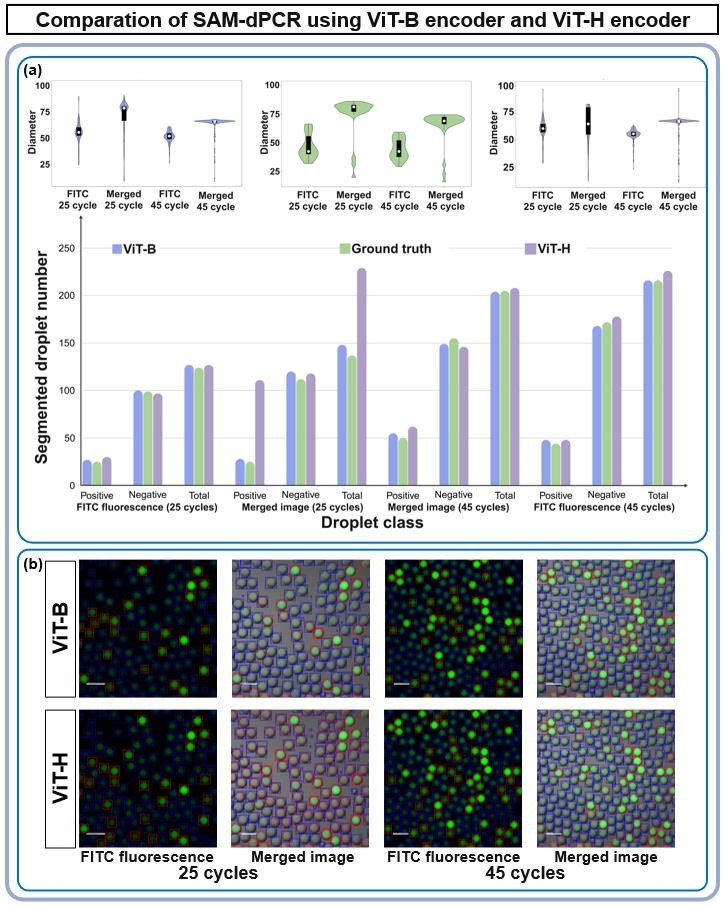


**Figure S9. Comparative assessment of SAM-dPCR with ViT-B and ViT-H encoders.** (a) Both encoders perform comparably in droplet segmentation and size determination, while ViT-B surpasses ViT-H in classification accuracy. (b) Representative images reveal that the ViT-H encoder is more prone to fluorescence intensity sensitivity and recounting errors than the ViT-B encoder.


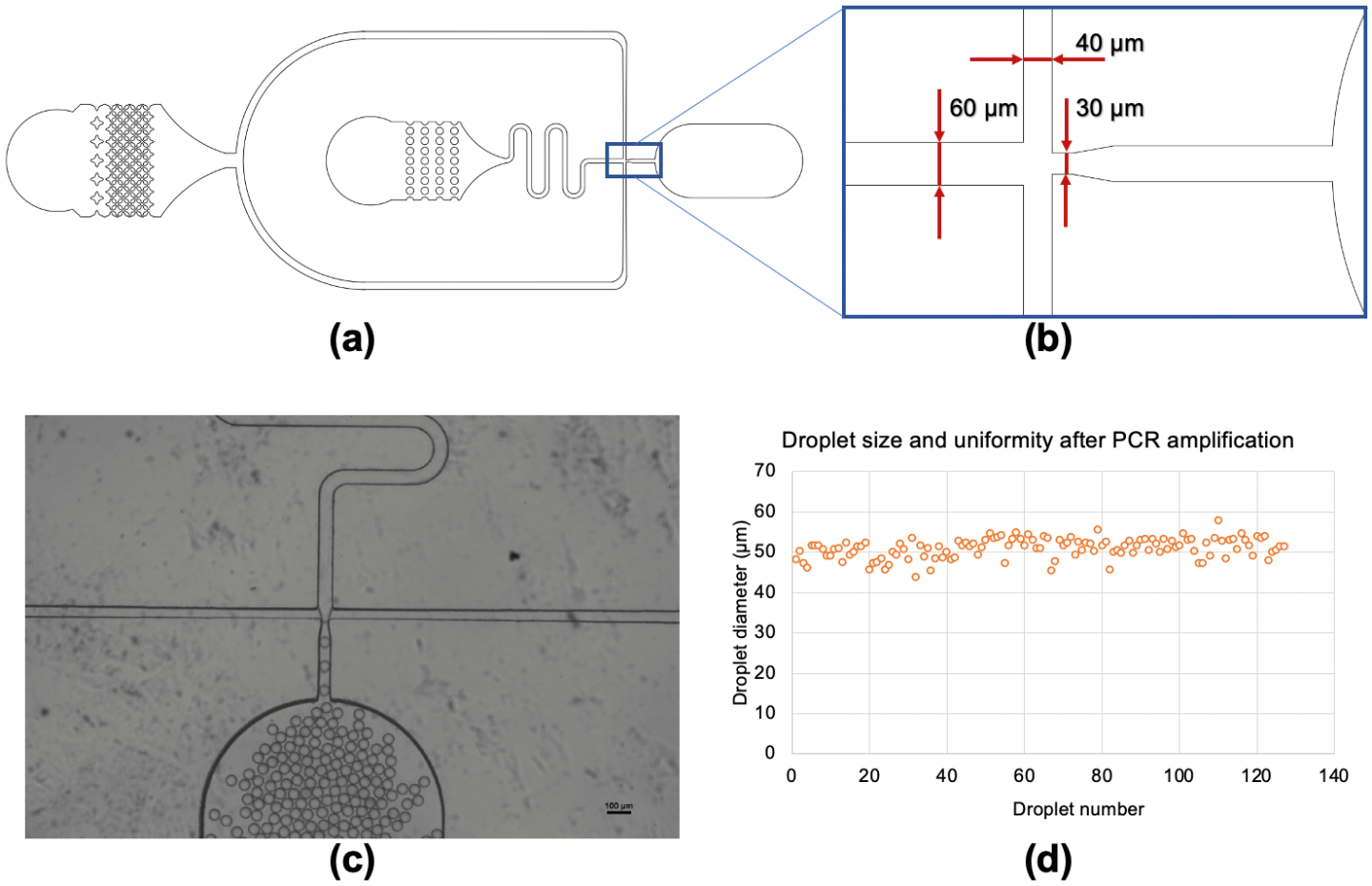


**Figure S10. Microfluidic chip design for uniform droplet generation and characterization.** (a) The flow-focusing microfluidic chip with a cross-sectional dimension of 30.0 μm in width and 38.5 μm in height was designed and fabricated to generate droplets. (b) Detailed illustration of the throat design in (a) with specific dimensions. (c) Bright-field microscopy image showing a representative frame of droplet generation (Scale bar: 100 μm). The monodisperse droplets were generated using the custom-designed microfluidic chip mentioned in (a). (d) Measurement of droplets after the PCR thermal amplification process. The droplets were stored at 4°C for at least 48 hours.
